# Global, regional and national burden of polycystic ovary syndrome: historical trends from 1990 to 2021 and projections to 2035

**DOI:** 10.3389/fendo.2026.1662823

**Published:** 2026-04-01

**Authors:** Hu Li, Zhaoyi Jing, Rui Li, Zheng Shen, Tianyu Bai, Qingyu Song

**Affiliations:** 1 Department of Acupuncture-Moxibustion and Tuina, Shandong Provincial Third Hospital, Shandong University, Jinan, China; 2 The First Clinical College, Shandong university of traditional Chinese medicine, Jinan, China; 3 College of Acupuncture and Massage, Shandong university of traditional Chinese medicine, Jinan, China; 4 The Seventh Clinical College, Shanghai University of Traditional Chinese Medicine, Shanghai, China

**Keywords:** decomposition analysis, frontier analysis, global disease burden, inequality analysis, polycystic ovary syndrome, socio-demographic index

## Abstract

**Background:**

This study aims to deepen the understanding and assessment of the global burden of polycystic ovary syndrome (PCOS), providing a basis for policy-making and resource allocation to help achieve global health goals.

**Methods:**

This study focused on analyzing the disease burden of PCOS at the global, regional, and national levels, considering age factors and predicting trends up to 2035. This study also involved cross-national inequality analysis, frontier analysis, and decomposition analysis.

**Results:**

The burden of PCOS is increasing, with the age of onset concentrated in the 10–19 age group, while the number of cases and DALYs is predominantly in the 15–49 age group. Countries with a higher Socio-demographic Index (SDI) bear a heavier burden of PCOS, effectively controlling its growth trend. Inequality persists. Population growth and epidemiological changes are driving the increase in the burden of PCOS. By 2035, the burden of PCOS is projected to continue increasing.

**Conclusion:**

The global burden of PCOS shows an overall increasing trend, with significant differences among different regions and countries. Greater support is needed for low SDI to standardize diagnostic criteria, raise public health awareness, and better protect women’s health.

## 1 Introduction

Polycystic Ovary Syndrome (PCOS) is a common endocrine disorder in women of reproductive age, characterized by irregular menstruation and hyperandrogenism (1, 2). In 2021, the global standardized prevalence rate (ASPR) among women of reproductive age was as high as 3364.53 cases per 100,000 people (95% UI: 2395.08 - 4681.81) (3).Although the exact cause of PCOS is not fully understood, research indicates that it is closely related to genetic and environmental factors, with diet and lifestyle also playing a role (4). PCOS is a major cause of female infertility (5) and is associated with increased risks of insulin resistance, obesity, metabolic disorders, and cardiovascular diseases (6–8). Additionally, compared to non-PCOS individuals, patients with PCOS are more likely to experience anxiety, depression, low self-esteem, and decreased quality of life (9, 10). This not only affects the health of patients but also increases the economic burden on healthcare systems (11, 12). In 2020, estimated healthcare expenditures in the United States due to PCOS-related pregnancy complications and long-term diseases were approximately $43 billion (11). Therefore, considering the significant impact of PCOS on economic systems, individual health, and public health, conducting in-depth research on its epidemiological characteristics, identifying relevant influencing factors, and providing policy recommendations for disease screening and resource allocation are of utmost importance.

PCOS has become a significant issue affecting the health of women worldwide, presenting differentiated challenges in different regions and populations (13, 14). The diagnosis of PCOS is highly complex and challenging primarily because it requires ruling out other diseases that may cause similar symptoms, leading to underdiagnosis or delays in diagnosis (15–17).An estimated 70% of people with PCOS are undiagnosed meaning that the financial, personal and societal burden of this disorder are even higher than currently understood (18). This is especially true in low Socio-demographic Index (SDI) with limited medical resources and diagnostic technologies, where underdiagnosis not only affects patient health but also exacerbates the burden on the healthcare system (19, 20). Therefore, gaining a deeper understanding of the differences in disease burden across countries with different economic levels is crucial for improving the efficiency and equity of global healthcare systems. By allocating resources effectively, it is possible to improve the diagnosis and management of PCOS, thereby alleviating the current unfavorable trends.

Social changes and economic development have led to significant shifts in the global population structure (21). From 1990 to 2019, the global population increased by 43% (21, 22). This growth necessitates adjustments in national healthcare systems to address the impact of population growth on the health sector (21). However, the specific effects of global population growth on the burden of PCOS have not been systematically studied. This research gap may limit the ability of the World Health Organization and national governments to effectively address the increased health demands resulting from population growth when formulating and adjusting health policies. Moreover, an in-depth exploration of the epidemiological characteristics of PCOS globally and predicting its future trends holds significant potential value for disease prevention.

## 2 Method

The objective of this study is to utilize the GBD statistical model to systematically describe the disease burden of PCOS in 2021 using the GBD statistical model, processing and analyzing data from global, regional, and national levels, and evaluating the temporal trends and epidemiological characteristics of PCOS. We also focus on age factors, as well as the association between PCOS and socio-economic status, conducting the first analysis of transnational inequalities in PCOS and frontier analysis, assessing the impacts of aging, population growth, and epidemiological changes on PCOS, and predicting trends up to 2035.

### 2.1 Data acquisition

This epidemiological study utilized retrospective data extracted from the Global Burden of Diseases (GBD) 2021 database, managed by the Institute for Health Metrics and Evaluation (IHME). The GBD database provides comprehensive population and epidemiological information spanning 1990 to 2021, covering 204 countries and territories. Data sources include censuses, household surveys, and disease registries. The GBD modeling framework handles missing data through spatiotemporal Gaussian process regression and covariate-based imputation in the DisMod-MR 2.1 Bayesian meta-regression tool. PCOS cases were identified using ICD-10 diagnosis codes (E28.2).The diagnostic criteria used by experts include those from the National Institutes of Health, the Rotterdam criteria, and the Androgen Excess Society. In GBD 2021, PCOS cases were identified based on any of these three diagnostic approaches. This epidemiological study utilized retrospective data extracted from the Global Burden of Diseases (GBD) 2021 database, managed by the Institute for Health Metrics and Evaluation (IHME). The GBD database provides comprehensive population and epidemiological information spanning 1990 to 2021, covering 204 countries and territories. Data sources include censuses, household surveys, and disease registries. The GBD modeling framework handles missing data through spatiotemporal Gaussian process regression and covariate-based imputation in the DisMod-MR 2.1 Bayesian meta-regression tool.

### 2.2 Research dimensions

The Socio-demographic Index (SDI) is a composite measure reflecting the health-related development status of a nation or territory (23, 24).The SDI is a composite metric developed by the Institute for Health Metrics and Evaluation (IHME) for the Global Burden of Disease (GBD) studies, ranging from 0 to 1. It integrates factors such as fertility, educational attainment, and economic prosperity. Based on their SDI values, countries and territories are stratified into five distinct development levels: Low (<0.45), Low-middle (≥0.45 to<0.61), Middle (≥0.61 to<0.75), High-middle (≥0.75 to<0.90), and High (≥0.90). Falling into a specific level indicates a country’s relative position in the global spectrum of socio-economic progress; for instance, a High SDI classification implies advanced educational attainment, lower early fertility, and higher economic prosperity, often correlating with better healthcare infrastructure, whereas a Low SDI classification suggests limited resources, lower education, and higher fertility, which often correlates with challenges in diagnosis and resource allocation. This stratification allows for a nuanced interpretation of how disease burden shifts across different stages of economic and social transition, highlighting disparities that aggregate global data might obscure.

### 2.3 Statistical analysis

This study evaluated incidence, prevalence, and DALYs rates using the Age-Standardized Rate (ASR) and its corresponding 95% Uncertainty Interval (UI), with the 95% UI being used to reflect the reliability of the data and the robustness of the model (25). The ASR denotes an estimate per 100,000 people, and the use of the ASR can facilitate scientific comparisons between populations, despite differences in age distribution and population size across populations, thereby improving the precision of population comparisons. These rates are derived from the following formula:


$$\text{ASR=}\frac{\sum_{i=1}^{\text{A}}{\text{a}}_{\text{i}}{\text{w}}_{\text{i}}}{\sum_{i=1}^{\text{A}}{\text{w}}_{\text{i}}}\times 100,000$$

Where A denotes the number of age groups, i denotes the ith age group, ai denotes the rate to be standardized, and wi denotes the number of standardized population of the same age. This study adopted the GBD World Standard Population as the reference to ensure international comparability of the results. The GBD standard population is based on the average age structure of countries globally, weighted by population size, making it suitable for cross-national and temporal comparisons.

Estimated Annual Percentage Change (EAPC) to analyze the trend over time was calculated from the regression model: Y is the natural logarithm of the ASR, α is the intercept, β is a variable that determines the positive or negative trend of the ASR, X denotes the calendar year, and ϵ is the error term (26). The EAPC and its 95% Confidence Interval (CI) are also derived from the model and calculated as EAPC=100*(exp (β);-1).

The Joinpoint regression model was employed to analyze temporal trends by segmenting the study period based on the distribution characteristics of the disease (27). Log-linear models were applied for segmented regression analysis. The grid search method identified all potential joinpoints, calculated the corresponding mean squared errors (MSE), and selected the joinpoint configuration with the minimal MSE as the optimal solution. A Monte Carlo permutation test was used to determine the optimal number of joinpoints, allowing for 0 to 5 joinpoints (28). The significance level was set at P< 0.05, with Bonferroni correction applied to control for multiple testing. The Average Annual Percent Change (AAPC) and its 95% confidence interval (CI) were calculated to characterize overall trend patterns. A statistically significant AAPC greater than zero (P< 0.05) indicates an increasing trend during the specified period.

To assess inequalities in PCOS burden across nations and territories, two key metrics endorsed by the World Health Organization were employed: the Slope Index of Inequality (SII) and the Concentration Index for Health Equity (CIX) (29). These measures provide a comprehensive assessment of both absolute and relative health inequalities.

Decomposition analysis quantified the relative contributions of population growth, aging, and epidemiological changes (including factors such as urbanization and lifestyle shifts) to increases in incidence, prevalence, and DALYs attributable to PCOS (30).The change in case numbers was decomposed using the formula: Total change = Change (population growth) + Change (aging) + Change (epidemiological change). Through decomposition of these contributions, the approach elucidated key drivers underpinning the rising burden of PCOS.

Frontier analysis assessed the unrealized potential for reducing PCOS burden relative to a socioeconomic performance benchmark (31). The health achievement framework1 was operationalized by defining the frontier as the lowest observed age-standardized rates for a given SDI level globally, establishing an evidence-based benchmark for minimal disease burden attainable at that development stage. Countries were stratified by their discrepancy from this frontier, prioritizing those with the largest absolute reductions achievable should they attain frontier-level performance. The frontier was defined as the lowest observed age-standardized rate at a given SDI level globally, establishing an evidence-driven benchmark representing the minimum disease burden achievable by the best-performing health systems at that level of socioeconomic development. A quantile regression approach at the 5th percentile was employed to construct a smooth frontier function across the SDI spectrum, ensuring robustness against isolated data points.

The BAPC model is a widely utilized method in epidemiology and biostatistics for analyzing temporal trends in incidence, prevalence, and DALYs (29). This approach integrates sample information with prior knowledge to derive unique parameter estimates, yielding robust and reliable results. In this study, the BAPC software package was employed to project trends in the prevalence and YLDs attributable to PCOS from 2022 to 2035. The insights generated by this modeling approach can inform the development of effective public health policies aimed at guiding prevention and management strategies for PCOS.

All p-values were two-sided and p<0.05 was considered statistically significant. all statistical analyses and data visualizations were performed using R (version 4.4.2) and JD_GBDR (V2.37, JingdingMedical Technology Co., Ltd.).

## 3 Results

### 3.1 Disease burden of PCOS

#### 3.1.1 Global trends

**Figure 1:**
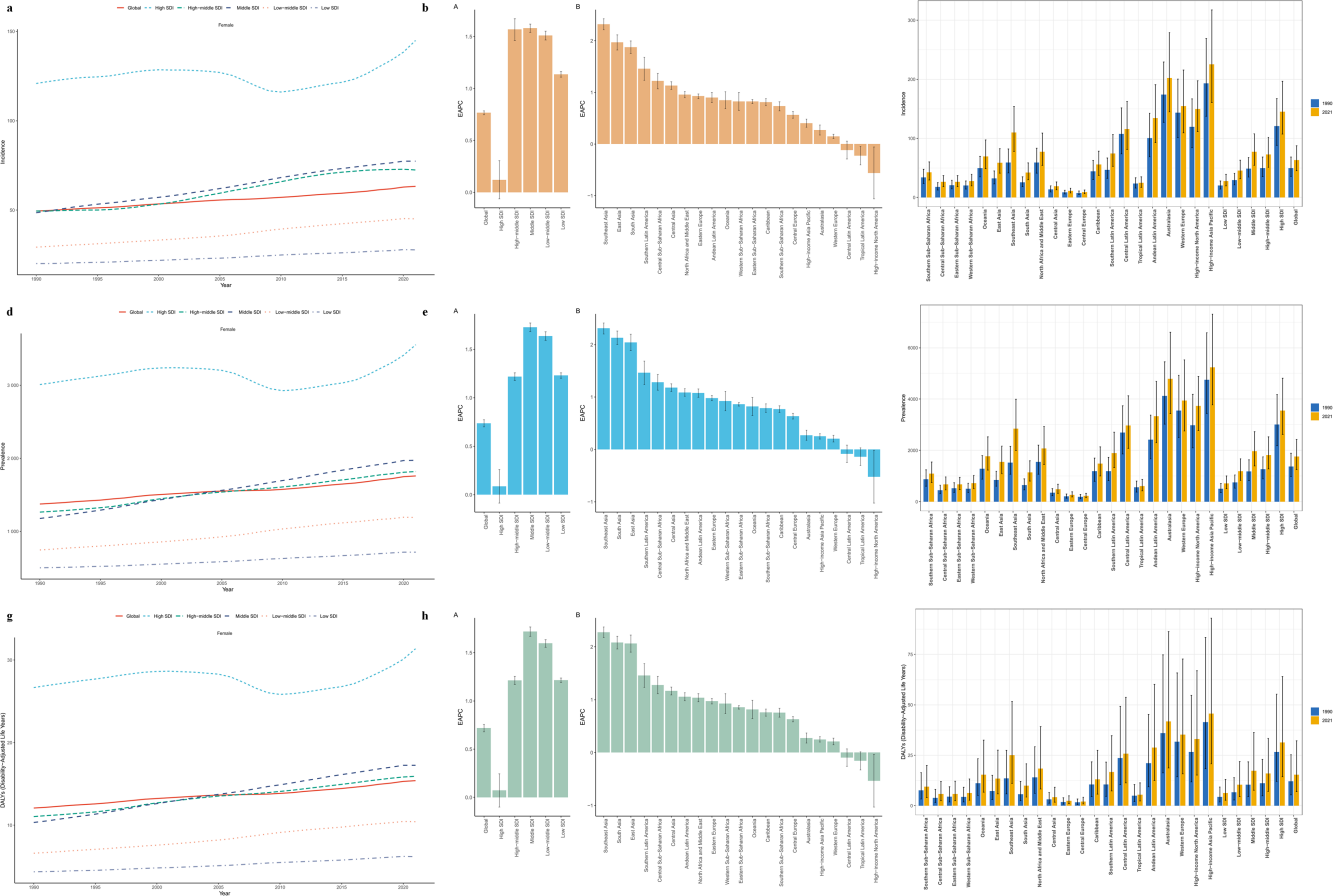
Changes in the burden of polycystic ovary syndrome and a comparison between 1990 and 2021. **(a–c)** Age-standardized incidence rate; **(d–f)** Age-standardized prevalence rate; **(g–i)** Age-standardized DALYs rate.

In 2021, the number of cases of PCOS globally increased significantly from 36,651,157 cases in 1990 (95% UI: 26,227,943–50,603,930) to 69,473,252 cases in 2021 (95% UI: 49,531,420–95,724,479). The age-standardized prevalence rate (ASPR) was 1,757.83 cases per 100,000 people (95% UI: 1,253.36–2,421.26) in 2021, with an estimated annual percentage change (EAPC) of 0.74% (95% CI: 0.70–0.77) from 1990 to 2021.In 2021, the number of new PCOS cases was 2,301,506 (95% UI: 1,655,989–3,167,178), and the age-standardized incidence rate(ASIR)increased from 49.45 cases per 100,000 (95% UI: 35.57–68.45) to 63.26 cases per 100,000 (95% UI: 45.41–87.28) with an EAPC of 0.77 (95% CI: 0.75–0.79). Over the 32-year period, the global DALYs rate increased by 0.72 (95% CI: 0.68–0.76), with a total of 607,757 DALYs in 2021 (95% UI: 272,745–1,268,607) and an age-standardized DALYs rate(ASDR)of 15.40 per 100,000 people (95% UI: 6.91–32.13). (**Table 1**, **Supplementary Table 1**, **Figure 1**).

**Table 1:** The number of disease burden cases of polycystic ovary syndrome in 2021, along with ASR and EAPC.

Characteristics	Incidence(95% uncertainty interval)			Prevalence(95% uncertainty interval)			DALYs(95% uncertainty interval)		
	Cases,2021	ASIR,2021	EAPC 1990-2021	Cases,2021	ASPR,2021	EAPC 1990-2021	Cases,2021	ASDR,2021	EAPC 1990-2021
Global	2301505.64(1655989.24,3167177.81)	63.26(45.41,87.28)	0.77(0.75,0.79)	69473252.37(49531420.00,95724479.23)	1757.83(1253.36,2421.26)	0.74(0.70,0.77)	607756.87(272745.15,1268607.22)	15.40(6.91,32.13)	0.72(0.68,0.76)
SDI level									
High SDI	494212.24(367071.51,670948.04)	144.89(107.28,196.81)	0.12(-0.06,0.30)	17573919.77(12981150.82,23876520.56)	3554.29(2624.21,4816.07)	0.09(-0.09,0.26)	154313.26(70664.08,314967.06)	31.37(14.37,64.00)	0.07(-0.10,0.25)
High-middle SDI	305809.98(216061.40,424907.89)	72.52(51.14,101.53)	1.57(1.46,1.67)	11180894.89(7874794.87,15590881.41)	1817.62(1277.35,2529.47)	1.22(1.18,1.26)	97184.50(43255.09,205403.82)	15.91(7.10,33.31)	1.21(1.17,1.25)
Middle SDI	812668.96(572963.70,1126099.74)	77.38(54.44,107.63)	1.58(1.54,1.62)	24613369.91(17452531.61,34074253.70)	1971.07(1395.79,2724.23)	1.73(1.68,1.77)	214898.85(95592.62,450661.83)	17.26(7.68,36.24)	1.72(1.67,1.77)
Low-middle SDI	481689.01(338285.68,670310.33)	45.20(31.82,62.95)	1.51(1.47,1.55)	12118405.37(8434180.98,17027257.26)	1188.74(828.69,1668.94)	1.64(1.59,1.68)	106509.68(46621.43,223924.95)	10.43(4.56,21.89)	1.60(1.56,1.64)
Low SDI	205458.30(144223.85,291379.81)	27.79(19.81,38.87)	1.14(1.11,1.16)	3938251.29(2745129.91,5589751.24)	714.31(504.52,1007.40)	1.23(1.20,1.26)	34425.20(14799.71,72773.06)	6.20(2.67,13.05)	1.21(1.19,1.24)
GBD Region									
Andean Latin America	42634.55(29473.44,60429.45)	134.14(92.65,190.70)	0.90(0.80,1.00)	1172864.51(808337.39,1649165.85)	3333.66(2301.61,4690.91)	1.08(1.00,1.15)	10129.25(4423.90,21176.60)	28.78(12.57,60.15)	1.06(0.98,1.13)
Australasia	21492.87(15427.88,29606.77)	202.25(145.07,278.86)	0.27(0.18,0.37)	701617.99(500341.02,974912.97)	4786.95(3430.23,6607.27)	0.27(0.18,0.37)	6103.56(2756.51,12632.80)	41.77(18.75,86.35)	0.28(0.18,0.37)
Caribbean	11962.48(8144.39,16730.14)	55.61(37.61,78.19)	0.81(0.74,0.88)	360981.83(245137.34,520033.49)	1485.97(1008.31,2136.92)	0.77(0.71,0.83)	3161.50(1381.53,6647.55)	13.03(5.70,27.37)	0.76(0.69,0.82)
Central Asia	8122.32(5654.13,11355.50)	18.79(13.06,26.29)	1.13(1.06,1.20)	237958.89(163368.39,333464.66)	483.56(330.71,678.72)	1.18(1.11,1.25)	2079.36(886.90,4469.52)	4.24(1.81,9.10)	1.16(1.09,1.24)
Central Europe	3175.48(2221.05,4441.04)	8.97(6.22,12.67)	0.57(0.50,0.63)	119149.23(82050.44,168514.00)	228.93(157.78,326.20)	0.64(0.58,0.69)	1032.65(442.86,2156.63)	2.00(0.86,4.17)	0.63(0.58,0.68)
Central Latin America	143013.73(100629.38,200290.73)	115.89(81.09,162.25)	-0.11(-0.29,0.06)	4072957.18(2852240.56,5662452.40)	2967.64(2076.22,4124.04)	-0.08(-0.25,0.08)	35314.12(15577.76,73735.75)	25.74(11.36,53.72)	-0.10(-0.26,0.07)
Central Sub-Saharan Africa	23871.94(16637.67,33976.02)	26.20(18.46,36.97)	1.22(1.07,1.37)	444120.79(305191.60,640542.02)	673.86(465.36,968.69)	1.28(1.13,1.43)	3861.86(1673.68,7930.54)	5.82(2.51,12.02)	1.28(1.12,1.44)
East Asia	268458.51(190157.13,373877.35)	58.63(41.20,82.32)	1.97(1.82,2.11)	10490358.54(7423407.50,14808757.10)	1548.43(1085.52,2170.68)	2.04(1.89,2.20)	89991.43(39441.80,185701.88)	13.38(5.91,27.61)	2.06(1.90,2.22)
Eastern Europe	7596.11(5457.51,10592.63)	10.86(7.64,15.38)	0.93(0.88,0.97)	265678.69(185374.43,381762.99)	264.99(182.67,381.43)	0.98(0.93,1.03)	2323.67(970.02,4890.37)	2.35(0.97,4.92)	0.97(0.92,1.02)
Eastern Sub-Saharan Africa	76605.14(53945.13,108540.53)	26.26(18.57,36.96)	0.82(0.79,0.86)	1438942.37(1001296.83,2063339.49)	669.17(472.35,949.85)	0.86(0.83,0.89)	12497.08(5380.70,26310.19)	5.77(2.49,12.17)	0.85(0.82,0.89)
High-income Asia Pacific	107170.54(75693.68,152114.34)	225.11(160.59,317.34)	0.40(0.33,0.48)	4104982.68(2922772.61,5775182.08)	5237.62(3779.21,7307.15)	0.25(0.20,0.30)	35520.50(16037.31,72064.21)	45.62(20.81,92.93)	0.25(0.20,0.30)
High-income North America	203413.49(150004.56,271137.80)	149.79(111.27,197.64)	-0.56(-1.06,-0.06)	6362238.32(4742961.86,8324162.21)	3729.48(2777.89,4876.97)	-0.52(-1.02,-0.02)	56162.19(25718.34,113710.16)	33.02(15.20,66.90)	-0.53(-1.03,-0.03)
North Africa and Middle East	242427.05(171058.09,342784.62)	77.15(54.45,109.17)	0.96(0.90,1.02)	6673431.49(4672056.28,9434543.48)	2075.28(1453.41,2932.69)	1.09(1.02,1.16)	59116.30(26476.78,125706.81)	18.40(8.24,39.14)	1.04(0.96,1.11)
Oceania	5300.91(3692.06,7439.78)	69.33(48.42,97.52)	0.85(0.69,1.01)	124484.33(86570.21,177516.43)	1772.65(1232.67,2531.47)	0.82(0.65,0.99)	1083.54(471.33,2285.57)	15.41(6.71,32.47)	0.82(0.64,0.99)
South Asia	416258.43(299377.40,573874.15)	42.08(29.94,58.32)	1.88(1.76,2.00)	11291117.08(7950085.92,15832639.77)	1135.87(799.70,1591.05)	2.13(2.00,2.26)	98946.93(43231.49,207059.23)	9.94(4.34,20.79)	2.08(1.96,2.20)
Southeast Asia	357262.30(254498.65,495461.45)	110.11(77.87,153.89)	2.32(2.21,2.43)	10520027.69(7378813.87,14809823.50)	2842.65(1993.15,3997.51)	2.31(2.20,2.42)	92605.68(41149.03,191024.08)	25.06(11.10,51.68)	2.28(2.18,2.38)
Southern Latin America	21574.43(15329.63,30970.01)	74.53(52.77,106.57)	1.46(1.23,1.68)	667604.83(469021.54,956761.41)	1892.50(1326.86,2707.18)	1.46(1.24,1.69)	5893.54(2574.80,12247.50)	16.74(7.33,34.66)	1.46(1.24,1.68)
Southern Sub-Saharan Africa	18377.82(12888.44,26037.99)	42.33(29.66,59.97)	0.73(0.64,0.82)	480389.59(328945.22,679589.28)	1094.76(749.33,1548.05)	0.79(0.71,0.87)	4157.74(1795.14,8746.01)	9.47(4.10,19.92)	0.75(0.67,0.84)
Tropical Latin America	23769.11(16611.45,32960.09)	24.90(17.12,35.01)	-0.22(-0.40,-0.05)	746471.68(514676.97,1057462.85)	610.00(419.25,869.52)	-0.14(-0.31,0.03)	6557.65(2825.05,13830.99)	5.38(2.33,11.29)	-0.16(-0.33,0.01)
Western Europe	206192.21(145653.51,286689.08)	154.64(109.19,215.84)	0.15(0.11,0.19)	7455929.27(5232259.44,10460556.82)	3942.92(2761.02,5529.65)	0.21(0.14,0.27)	66041.54(29857.20,136985.13)	35.16(15.84,72.77)	0.21(0.15,0.27)
Western Sub-Saharan Africa	92826.23(65226.48,132560.86)	27.80(19.81,39.19)	0.83(0.65,1.00)	1741945.38(1209889.21,2493010.70)	722.68(508.32,1026.25)	0.92(0.74,1.11)	15176.76(6521.52,32235.88)	6.24(2.69,13.23)	0.93(0.74,1.11)

#### 3.1.2 SDI regional levels

In 2021, the ASIR, ASPR, and ASDR of PCOS in the 5 SDI regions all showed increases compared to 1990. The high SDI region had the highest ASIR, ASPR, and ASDR, with values of 144.89 cases per 100,000 (95% UI: 107.28–196.81), 3,554.29 cases per 100,000 (95% UI: 2,624.21–4,816.07), and 31.37 cases per 100,000 (95% UI: 14.37–64.00), respectively. However, the high SDI region was the only SDI region that had growth rates below the global average, with EAPC of 0.12 (95% CI: -0.06–0.30), 0.09 (95% CI: -0.09–0.26), and 0.07 (95% CI: -0.10–0.25) for ASIR, ASPR, and ASDR respectively. The Middle SDI region had the highest growth rates with EAPC of 1.58 (95% CI: 1.54–1.62), 1.73 (95% CI: 1.68–1.77), and 1.72 (95% CI: 1.67–1.77) for ASIR, ASPR, and ASDR, respectively. This region also had the highest incidence of new cases of PCOS (812,669 cases), prevalence (24,613,370 cases), and DALYs (214,899 cases) among the 5 SDI regions. (**Table 1**, **Supplementary Table 1**, **Figure 1**).

#### 3.1.3 Changes in 21 geographical regions

**Figure 2:**
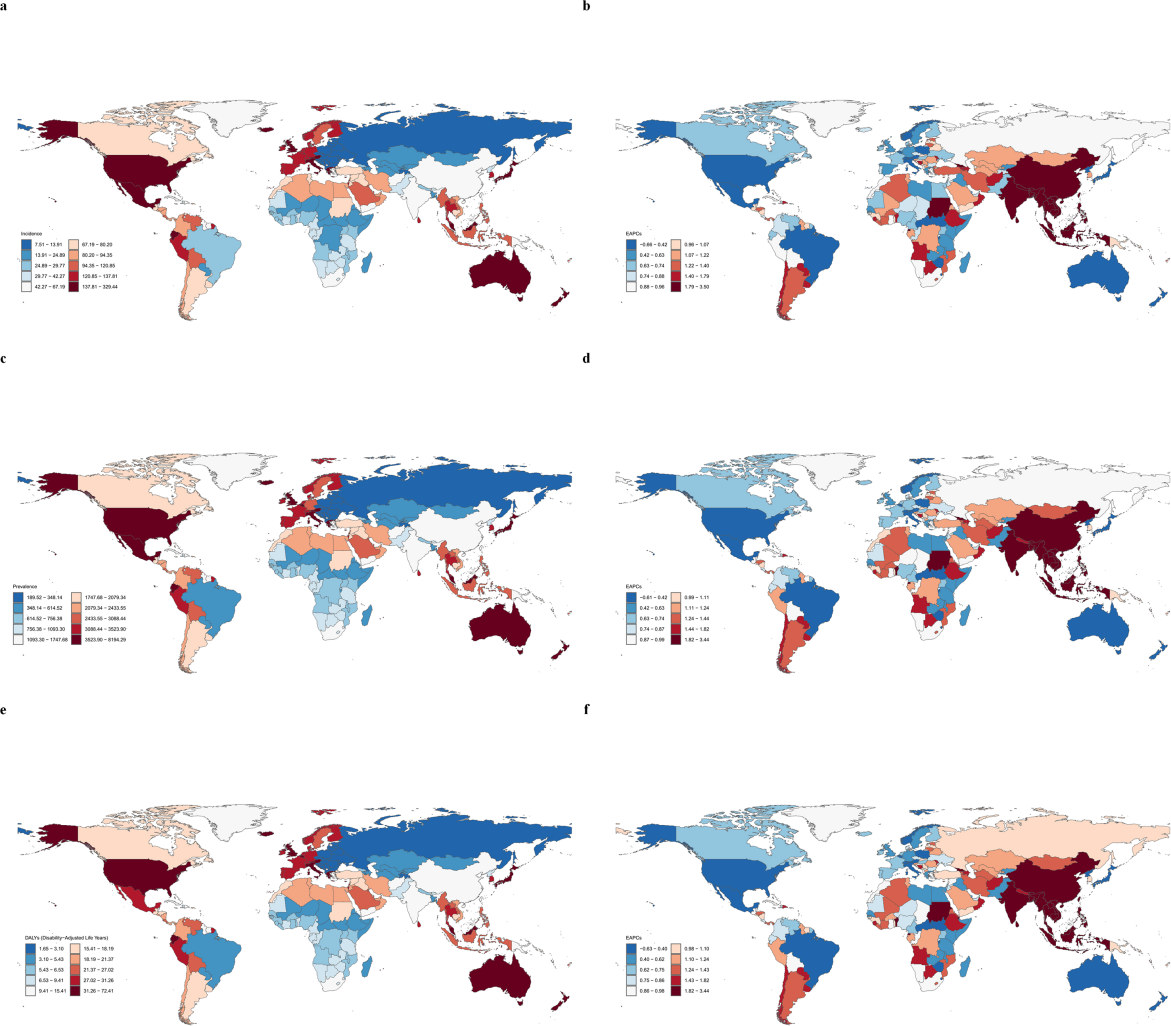
Global distribution of polycystic ovary syndrome burden in 2021. **(a, b)** Age-standardized incidence rate; **(c, d)** Age-standardized prevalence rate; **(e, f)** Age-standardized DALYs rate.

From 1990 to 2021, the ASIR, ASPR, and ASDR of PCOS generally showed an upward trend across most regions, with the exception of High-income North America, Tropical Latin America, and Central Latin America, which showed a downward trend. High-income North America had the greatest decrease, with EAPC of -0.56 (95% CI: -1.06–0.06) for ASIR, -0.52 (95% CI: -1.02–0.02) for ASPR, and -0.53 (95% CI: -1.03–0.03) for ASDR. In contrast, Southeast Asia had the highest increase, with EAPC of 2.32 (95% CI: 2.21–2.43), 2.31 (95% CI: 2.20–2.42), and 2.28 (95% CI: 2.18–2.38) for ASIR, ASPR, and ASDR, respectively. High-income Asia Pacific had the highest ASIR, ASPR, and ASDR values, and Central Europe had the lowest values. South Asia had the highest number of new cases (416,258 cases), prevalence (11,291,117 cases), and DALYs (98,947 cases) among the 21 regions, while Central Europe had the lowest (**Table 1**; **Supplementary Table 1**; **Figures 1**, **2**).

#### 3.1.4 Countries and territories

India reported the highest number of new PCOS cases, with 346,724 cases (95% UI: 248,264–476,794), while Tokelau had the fewest cases at 1 (95% UI: 1-1). China had the highest prevalence (10,077,520 cases, 95% UI: 7,120,017–14,249,277) and DALYs (86,443 cases, 95% UI: 37,911–178,421) among 204 countries. Italy had the highest ASIR (326.18 cases per 100,000), ASPR (8,113.16 cases per 100,000), and ASDR (71.69 cases per 100,000) values. Generally, the prevalence of PCOS increased in most countries, with Maldives showing a particularly significant increase with an EAPC of 3.41 (95% CI: 3.13–3.70). On the other hand, 8 countries showed a decrease in prevalence, with the United States having the largest decrease with an EAPC of -0.60 (95% CI: -1.14–0.06). Incidence rates and DALYs rates showed similar changing trends as prevalence (**Supplementary Table 2**; **Figure 2**).

### 3.2 Age distribution and temporal trends

**Figure 3:**
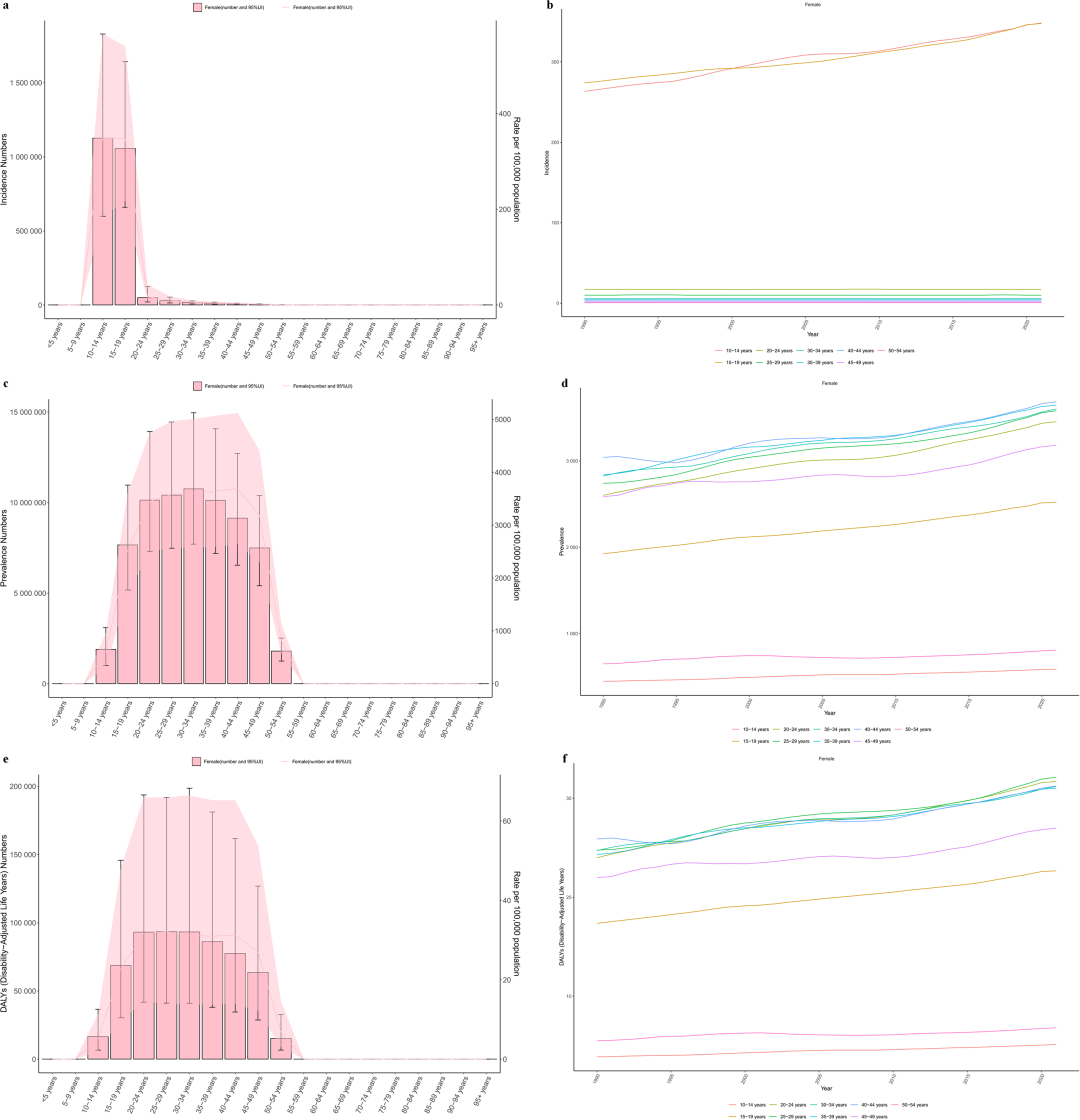
Age distribution and trend of the polycystic ovary syndrome burden in 2021. **(a,b)** Incidence; **(c, d)** Prevalence; **(e, f)** DALYs.

In 2021, the global incidence of PCOS was the highest in the 10–14 and 15–19 age groups, and it was on the rise. The incidence rates in other age groups remained relatively stable. The global number of PCOS cases and DALYs were mainly concentrated in the 15–49 age group, which also showed an increasing trend over time. Changes in other age groups were relatively minor (**Figure 3**).

### 3.3 Join-point regression analysis

**Figure 4:**
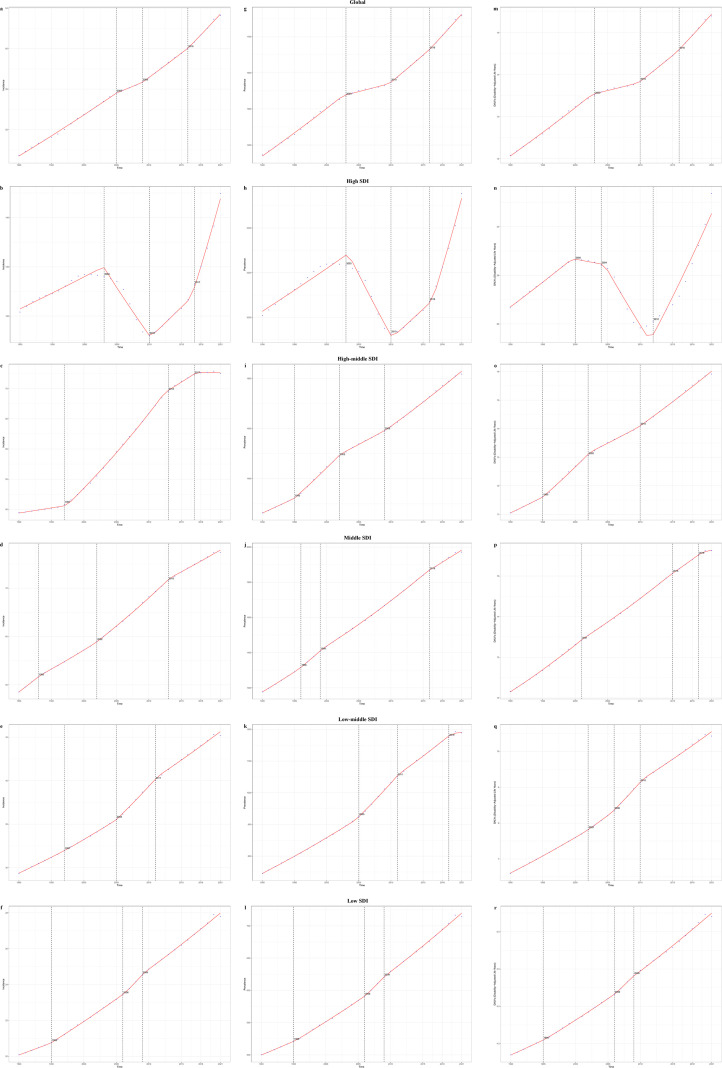
Join-point Regression Analysis of temporal trends in the burden of polycystic ovary syndrome from 1990 to 2021. **(a–f)** Age-standardized incidence rate; **(g–l)** Age-standardized prevalence; **(m–r)** Age-standardized DALYs rate.

From 1990 to 2021, the overall ASIR, ASPR, and ASDR of PCOS globally showed an increasing trend, with AAPC of 0.45 (95% CI: 0.45–0.46), 12.71 (95% CI: 12.53–12.88), and 0.11 (95% CI: 0.11–0.11) respectively. The trends in different disease burdens varied across different years, with the most significant increases in incidence rates, prevalence rates, and DALYs rates observed between 2016 and 2021, with APC of 1.10 (95% CI: 0.99–1.20, p<0.001), 1.15 (95% CI: 1.06–1.24, p<0.001), and 1.13 (95% CI: 1.04–1.21, p<0.001), respectively. (**Supplementary Table 3**; **Figure 4**). Join-point regression analysis revealed that high SDI regions showed a trend of decline followed by an increase in ASIR, ASPR, and ASDR. ASIR and ASPR showed declines from 2003 to 2010 with APC of -1.59 (95% CI: -1.77 to -1.40, p<0.001) and -1.77 (95% CI: -1.99 to -1.54, p<0.001) respectively, while ASDR had the most significant decrease from 2004 to 2012 with an APC of -1.65 (95% CI: -2.04 to -1.26, p<0.001). The highest increases were seen in ASIR (APC of 3.40 from 2017 to 2021, p<0.001), ASPR (APC of 3.09 from 2016 to 2021, p<0.001), and ASDR (APC of 2.00 from 2012 to 2021, p<0.001) during specific time periods. The other four regions showed continuous increases (**Supplementary Table 3**; **Figure 4**).

### 3.4 Correlation of SDI with regional and national PCOS disease burden and EAPC

We further explored the association between the SDI in 2021 and the burden of PCOS in different countries and regions globally. The results showed a positive correlation between SDI and age-standardized incidence rates in 21 regions (R = 0.4145, p< 0.001) and 204 countries (R = 0.448, p< 0.001). Similar correlations were observed for prevalence rates and DALYs rates with R values ranging from 0.4133 to 0.4546 (all p< 0.001). Additionally, a negative correlation was observed between EAPC and changes in age-standardized incidence rates, prevalence rates, and DALYs rates, with R values ranging from -0.16 to -0.16 (all p< 0.021). SDI was also correlated with EAPC with R values ranging from -0.15 to -0.16 (all p< 0.038) (**Supplementary Figure 2**).

### 3.5 Inequality analysis

**Figure 5:**
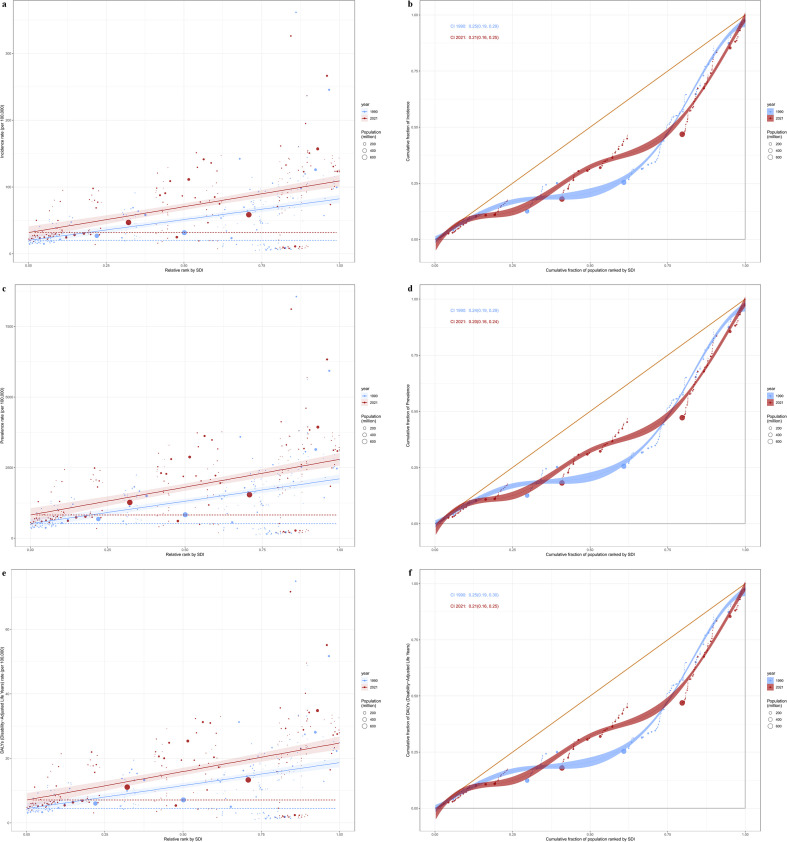
SDI-related health inequality regression curves and concentration curves for the global burden of polycystic ovary syndrome, 1990 and 2021. The health inequality regression curve on the left and the concentration curve on the right; **(a, b)** Age-standardized incidence rate; **(c, d)** Age-standardized prevalence rate; **(e, f)** Age-standardized DALYs rate.

We investigated the relationship between SDI and health inequalities and analyzed the correlation between the burden of PCOS and SDI levels, revealing the absolute and relative health inequalities at the SDI level. The study found that countries with higher SDI levels bore relatively higher burdens of ASIR, ASPR, and ASDR. Absolute health inequality in PCOS showed an increase from 1990 to 2021, but relative inequality showed a decrease (**Supplementary Table 5**, **Figure 5**).

### 3.6 Decomposition analysis

**Figure 6:**
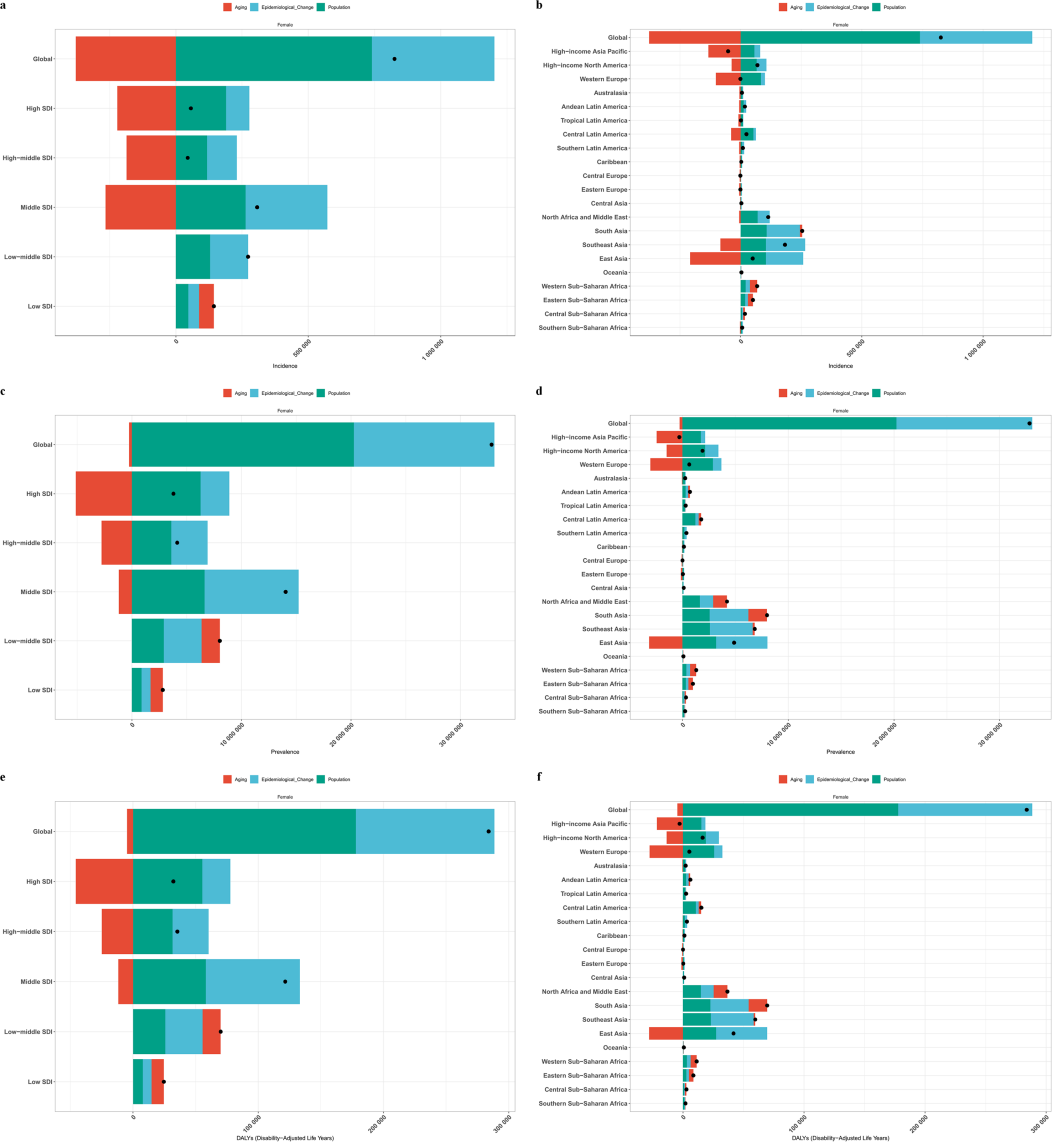
Key drivers of polycystic ovary syndrome burden at global and SDI levels from 1990 to 2021: population growth, ageing, and epidemiological changes. The black dots represent the sum of contributions to changes in all three factors. **(a, b)** incident cases, **(c, d)** prevalent cases, and **(e, f)** DALYs.

From 1990 to 2021, the increase in the number of PCOS cases globally was driven by population growth (61.71%) and epidemiological changes (39.08%), partially offset by aging (-0.79%). In the 5 SDI regions, the aging of low SDI and low-middle SDI regions contributed to an increase in the number of cases. However, in the 21 regions, the effects of aging in the high-income Asia Pacific outweighed population growth and epidemiological changes, resulting in a decrease in cases. The global increase in DALYs rates aligned with the patterns observed in incidence rates. Population growth and epidemiological changes drove the increases in the numbers of cases, prevalence, and DALYs globally and across the 5 SDI regions and 21 regions (**Figure 6**).

### 3.7 Frontier analysis

**Figure 7:**
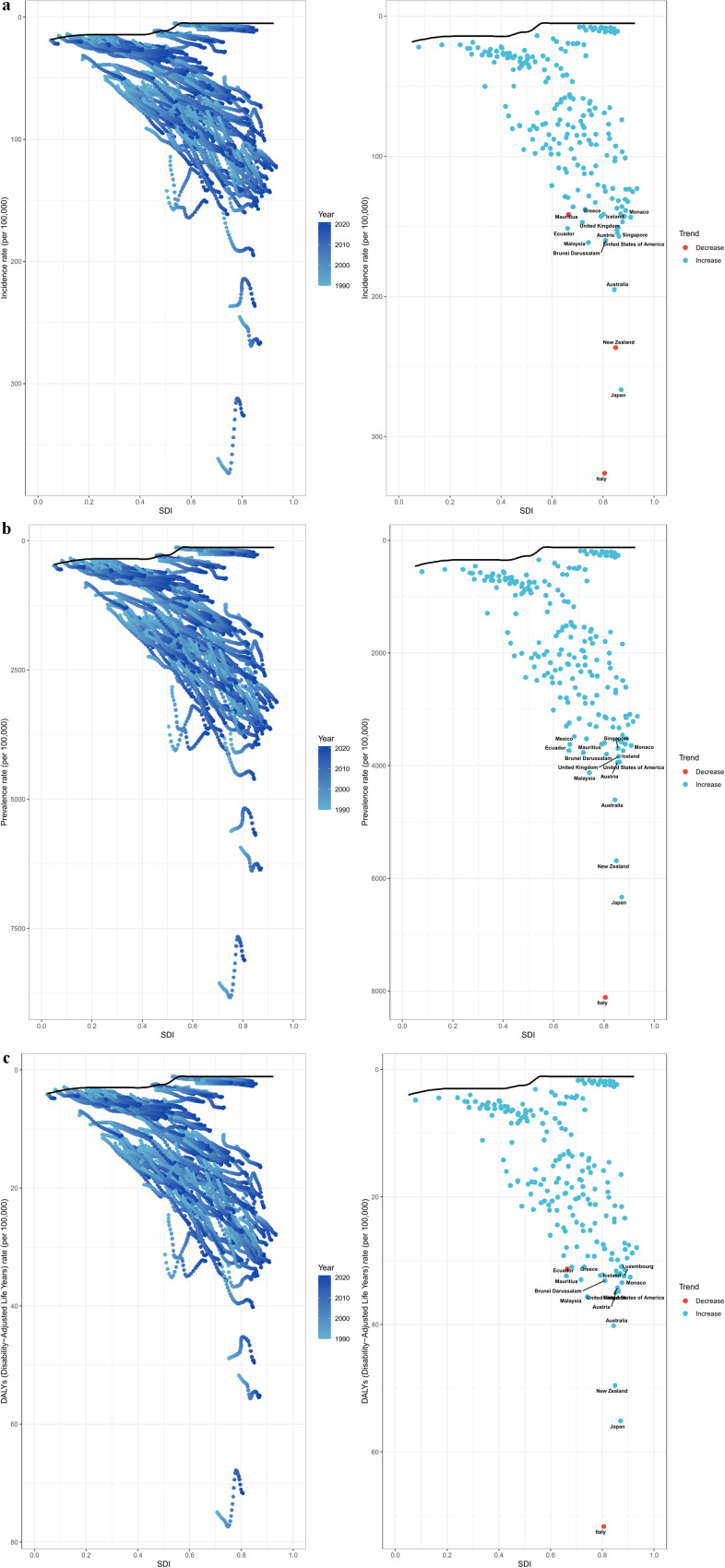
Frontier analysis involving SDI and polycystic ovary syndrome burden in 2021. Borders are marked in solid black, and countries and regions are represented by dots. The top 15 countries with the largest effective differences (with the highest border gaps) are marked in black. The red dots represent the reduction in the burden of polycystic ovary syndrome between 1990 and 2021. The blue dots indicate an increase in the burden of polycystic ovary syndrome over the same time period. **(a)** Age-standardized incidence rate; **(b)** Age-standardized prevalence rate; **(c)** Age-standardized DALYs rate.

Frontier analysis was conducted on ASIR, ASPR, and ASDR to explore the potential improvement space in the burden of PCOS in different countries based on their SDI. Countries with higher SDI (>0.80) like Italy, Japan, New Zealand, and Australia showed relatively higher effective differences in the burden of PCOS (**Figure 7**).

### 3.8 Forecasting trends in the disease burden of PCOS

**Figure 8:**
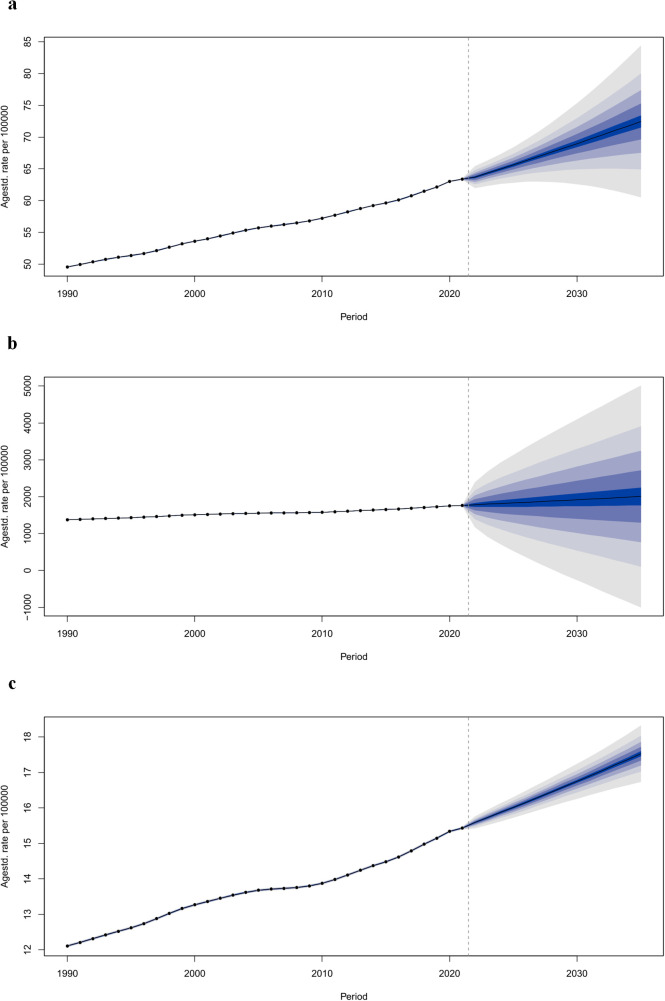
Trends in the burden of polycystic ovary syndrome: observed rates (1990–2021) and predicted rates (2022–2050). **(a)** Age-standardized incidence rate; **(b)** Age-standardized prevalence rate; **(c)** Age-standardized DALYs rate. The blue region in shows the upper and lower limits of the 95% UI.

We conducted future predictions and trend analyses on the ASIR, ASPR, and ASDR of PCOS. By 2035, the ASIR of PCOS is expected to be 72.46 per 100,000 people (95% UI: 58.28–86.64), the ASPR to be 2005.07 per 100,000 people (95% UI: -1572.04 to 5582.19), and the ASDR to be 17.53 per 100,000 people (95% UI: 16.59–18.47) (**Figure 8**).

The disease burden of PCOS is projected to continue to rise. Overall, the ASIR, ASPR, and ASDR of PCOS in all age groups are expected to increase continuously (**Supplementary Figures 3**-**S5**).

## 4 Discussion

Our research indicates that the disease burden of PCOS are all on the rise. The age group with the highest incidence is predominantly 10–19 years old, while the number of cases and DALYs are mainly concentrated in the 15–49 age group. Countries with higher SDI levels bear a heavier burden of PCOS, especially in high-income Asia Pacific, Australasia, and Western Europe, with Italy and Japan facing particularly severe situations. Inequality persists, and no country is at the forefront of reducing the burden of the disease. Population growth and epidemiological changes are driving an increase in the burden of PCOS. The disease burden of PCOS is projected to continue to rise through 2035.

Since 1990, the global burden of PCOS has continued to increase, with the burden being most significant in high SDI regions. This is closely related to the lifestyle and dietary habits in high SDI regions, where high-calorie, high-fat, and high-sugar dietary patterns are closely associated with obesity and insulin resistance (32, 33) both of which are important risk factors for PCOS (34, 35). Additionally, in high SDI regions, abundant medical resources and advanced diagnostic technologies allow for PCOS patients to be identified and diagnosed earlier and more accurately (13, 36). The increasing awareness of women’s health is also an important factor driving the rise in PCOS diagnosis rates (37). Meanwhile, in moderate SDI regions, the burden of PCOS is growing at a rapid pace, particularly in rapidly urbanizing Southeast Asia. In addition to the influence of lifestyle and medical resources (38, 39), factors such as a high prevalence of metabolic syndrome (40) and changes in population demographics are major reasons for the increasing disease burden in this region (41, 42). Additionally, the widespread use of the Rotterdam criteria (43) has expanded the diagnostic criteria for PCOS, to more patients being diagnosed. This has, to some extent, increased the global burden of the disease (44, 45).

Based on our analysis of age distribution, we found that the peak incidence of PCOS is mainly concentrated in the 10–19 age group. This is because adolescence is a key stage for the development of the female reproductive system, where various organ systems are still developing, hormone levels fluctuate significantly, and the regulation of the hypothalamic-pituitary-ovarian axis is not fully matured, making hormonal imbalances more likely to occur (8, 46, 47). Although PCOS has a higher incidence during adolescence, its disease burden is more significant in the 15–49 age group. Women in this age group are more prone to obesity and metabolic abnormalities, factors that further exacerbate the burden of PCOS (48, 49). Additionally, women of childbearing age seek medical attention more frequently due to their reproductive health needs and issues such as irregular menstruation, infertility, acne, etc. This behavior increases the diagnosis rate of PCOS (50, 51). PCOS is a chronic condition that requires long-term management starting from adolescence. It is recommended to reduce the intake of high-sugar, high-fat, and high-salt foods while increasing the consumption of dietary fiber, whole grains, and vegetables (52, 53). Additionally, patients should increase their physical activity, control weight, actively regulate menstrual cycles, and improve their status through medication or lifestyle changes. It is also important to undergo regular screenings to reduce the burden of the disease and improve long-term health outcomes (54, 55).

The COVID-19 pandemic has changed the global landscape of disease burden, but Join-point results reveal that the pandemic has not affected the trend of the disease burden of PCOS (56–58). An important turning point was in 2016 when there was a slight increase in the global disease burden of PCOS. This temporal association might reflect, in part, improved diagnostic ascertainment following the 2016 publication of the article “Clinical Practice. Polycystic Ovary Syndrome” (59), although other factors such as technological advances and changing lifestyles likely contributed concurrently. While the Rotterdam criteria are widely used in clinical practice, there are still differences in diagnostic criteria and practices among different countries and regions (60), it is necessary to conduct in-depth research on the clinical characteristics and pathogenesis of PCOS using big data and multi-omics technologies to improve diagnostic criteria. Additionally, there is a need for enhanced international collaboration to integrate diagnostic experiences from different countries and regions. Furthermore, tailored diagnostic guidelines should be developed for adolescent women and special populations (such as obese patients) to reduce underdiagnosis and delays in diagnosis, thereby better-safeguarding women’s health.

The SDI is positively correlated with the ASR of PCOS but negatively correlated with the EAPC. Uneven economic development has had a significant impact on the allocation of healthcare resources and access to medical services. In high SDI regions, advanced healthcare and quality health services have enhanced diagnostic capabilities, while better medical interventions and health awareness have controlled the growth of the disease burden of PCOS (61–63). However, low SDI regions with limited resources and inadequate medical services have not achieved the same level of improvement (64, 65). It is crucial to promote the fair distribution of global healthcare resources, strengthen support for low SDI regions, and improve basic healthcare infrastructure and medical services. This emphasizes the importance of elevating the SDI as a core objective of health policies.

Cross-national inequality analysis shows that the relative inequality of PCOS has decreased, mainly attributed to the widespread dissemination of medical technology, strengthened international cooperation (66, 67), increased public health awareness (37), and greater emphasis on the management of non-communicable diseases (68, 69). These factors have collectively promoted the global standardization of PCOS diagnosis and management. However, absolute health inequality is on the rise, reflecting unequal distribution of healthcare resources, disparities in status, and variations in health awareness and education levels. In the future, it is imperative to further strengthen international cooperation and policy support, increase investment in healthcare resources for low SDI regions, raise public health awareness, and effectively narrow the gap in global health inequality. Furthermore, PCOS is classified as a “common gynecological disease” and suffers from systematic neglect. This often leads to insufficient investment in research funds, uneven distribution of specialized medical resources, and a reduction in the priority given to public health policies (70, 71). This results in a health inequality phenomenon where “the disease is prevalent but the resource allocation is unequal”. Therefore, incorporating gender as the core dimension in health equity analysis is necessary to truly reveal and improve the long-overlooked health disparities of this group.

Although the SDI levels are similar, there are still significant differences in the burden of PCOS among different countries and regions. These differences cannot be simply explained by the SDI levels. Essentially, they are the concrete manifestations of multi-dimensional differences such as cognition, healthcare, culture, and genetics in a specific disease. Even if the SDI levels are similar, it does not mean that the distribution of medical resources is uniform. The differences in the density of specialists and the accessibility of examinations within a region will directly be reflected in the diagnosis rate of PCOS (72, 73); even if the same diagnostic criteria are used, the medical traditions and clinical practice habits in different regions will cause deviations in the implementation of the standards, resulting in uneven diagnosis rates and disease awareness levels (74); cultural and gender perspectives indirectly affect the disease burden through influencing patients’ medical-seeking behaviors (75, 76); differences in dietary structure, sedentary habits, and life rhythms will directly affect the occurrence and development of PCOS (77); in addition, the genetic background of specific races may increase the susceptibility to the disease (78, 79). Overall, these differences are the result of the joint action of multiple factors such as medical resource allocation, social culture, lifestyle, genetic susceptibility, and diagnostic practices. In the future, in-depth research on the challenges faced by countries with poor performance in PCOS management and addressing knowledge gaps may help alleviate the disease burden of PCOS.

Population growth and epidemiological changes are driving the increase in the global burden of PCOS. The expansion of the population directly results in an increase in the absolute number of disease burden, highlighting the importance of addressing population growth issues and suggesting it may be a potential strategy to alleviate the disease burden of PCOS in specific regions. Additionally, epidemiological changes are reflected in improvements in diagnostic criteria and lifestyle modifications. Due to variations in the demographic and epidemiological patterns of PCOS globally, it is evident that specific preventive and management measures for PCOS need to be developed for different regions, countries, and even on a global scale.

Based on the BAPC model’s predictions, the disease burden of PCOS is projected to continue rising until 2035. PCOS is a key area for public health intervention and is preventable. Future progress in the treatment and management of PCOS, healthcare reforms, and global public health initiatives may significantly alter the predicted developmental trend of PCOS.

Our study has certain limitations, with the data sources and quality in the GBD database are inconsistent, especially in economically underdeveloped areas, where inadequacies in the healthcare system may lead to potential misdiagnosis and underdiagnosis issues. Despite using complex statistical models to adjust data errors, this may still affect the comparability of the data. The DALYs provided by GBD 2021 may not fully reflect the specific impact of polycystic ovary syndrome (PCOS) on patients’ daily life functions and mental health. As a forward-looking suggestion, future research should incorporate quality-adjusted life years (QALYs) to better capture the multi-dimensional quality of life deficits associated with PCOS, including psychological distress, body image issues, and the stress related to infertility, thereby providing a more patient-centered perspective on the disease burden.

## 5 Conclusion

Between 1990 and 2021, the global burden of PCOs has shown an overall increasing trend. However, due to the unequal distribution of healthcare resources and lifestyle differences, significant differences exist among different regions and countries. Countries with higher SDI levels, although having a heavier burden of PCOS, have effectively controlled the growth trend, while low SDI regions have not achieved the same level of improvement. Future research should prioritize the establishment of a standardized global framework for the diagnosis and data reporting of PCOS, deeply explore the challenges faced by countries with poor management performance to fill knowledge gaps, and deepen research on the disease mechanism to develop more precise prevention and treatment methods; at the policy level, gender equality should be incorporated into the core dimension of global health equity, increase resource investment in low SDI countries, conduct standardized diagnosis training, and formulate differentiated regional prevention strategies to narrow the resource gap; in clinical practice, the early screening of high-risk populations aged 10–19 should be optimized, and standardized diagnostic guidelines based on age stratification (especially during puberty) and specific phenotypes (such as obese type) should be established to reduce missed diagnoses and misdiagnoses, promote the integration of lifestyle intervention, psychological support, and long-term metabolism in a multidisciplinary comprehensive management model, and implement full-cycle management from adolescence to reproductive age; and through strengthening public health education to enhance disease awareness, effectively curb the growth of PCOS burden, and promote the balanced improvement of global female health levels.

## Data Availability

The original contributions presented in the study are included in the article/**Supplementary Material**. Further inquiries can be directed to the corresponding authors.

## Supplementary material

Supplementary Figure 1 Correlation between ASR of polycystic ovary syndrome and SDI at the national and regional levels in 2021. **(a, b)** Age-standardized incidence rate; **(c, d)** Age-standardized prevalence rate; **(e, f)** Age-standardized DALYs rate.

Supplementary Figure 2 Correlation between Estimated Annual Percentage Change (EAPC) in 2021 and age-standardized rates of polycystic ovary syndrome and SDI. In the left figures, circles represent countries, while in the right figures,circles represent countries for which Human Development Index data is available. The size of each circle isproportional to the number of **(a)** incident cases, **(b)** prevalent cases, and **(c)** DALYs. The ρvalues and p-values were obtained from Pearson correlation analysis.

Supplementary Figure 3 Trends of age-standardized incidence rate of polycystic ovary syndrome across age groups: observed rates (1990–2021) and predicted rates (2022–2050).

Supplementary Figure 4 Trends of age-standardized prevalence rate of polycystic ovary syndrome across age groups: observed rates (1990–2021) and predicted rates (2022–2050).

Supplementary Figure 5 Trends of age-standardized DALYs rate of polycystic ovary syndrome across age groups: observed rates (1990–2021) and predicted rates (2022–2050).
