# Supplementary figures and images for: Global, regional and national burden of polycystic ovary syndrome: historical trends from 1990 to 2021 and projections to 2035
Source: Front Endocrinol (Lausanne). 2026 Apr 1;17:1662823. doi: 10.3389/fendo.2026.1662823 (PMC13079045; doi:10.3389/fendo.2026.1662823)

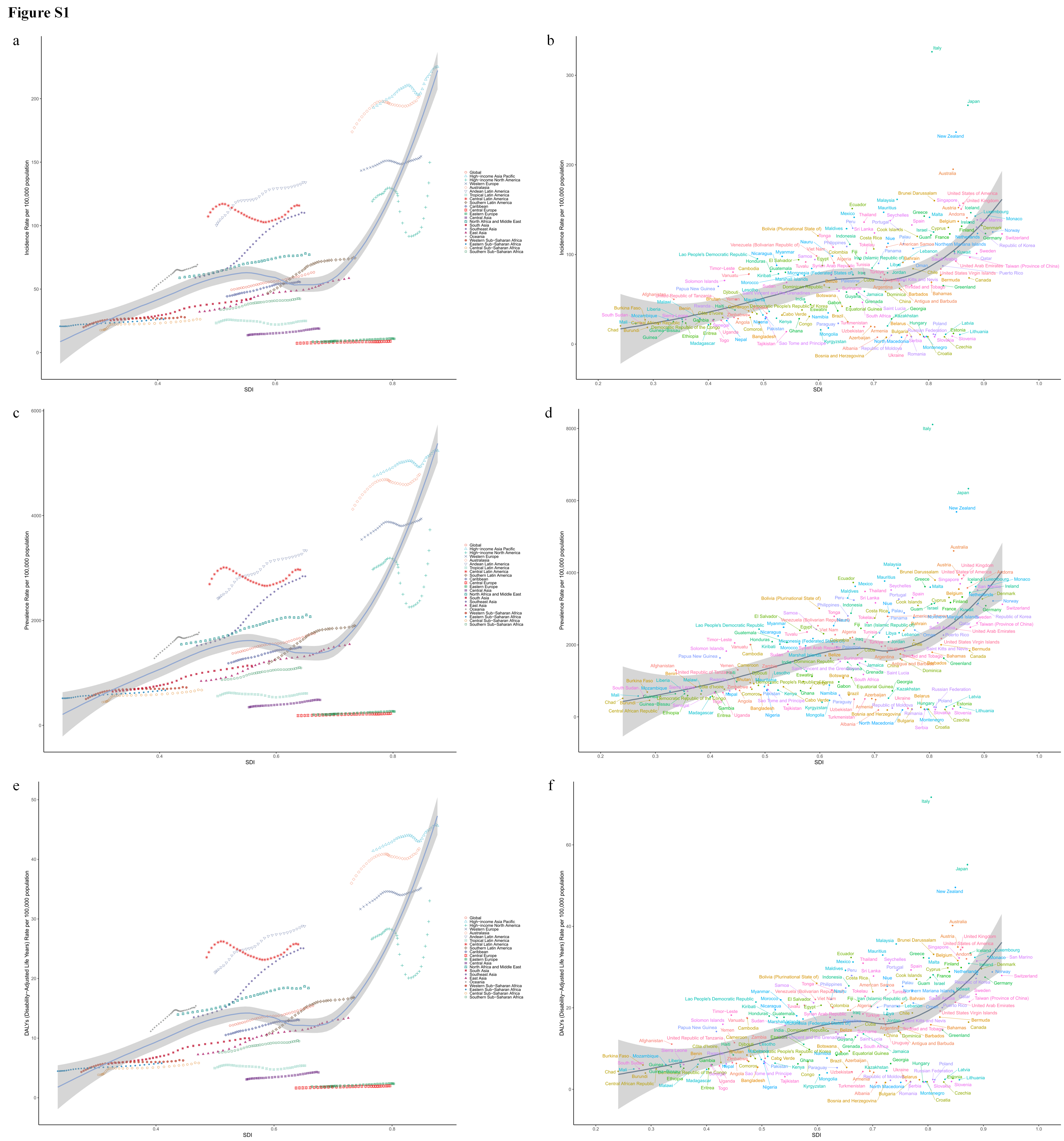

Supplement: Supplementary Figure 1 — Correlation between ASR of polycystic ovary syndrome and SDI at the national and regional levels in 2021. (a, b) Age-standardized incidence rate; (c, d) Age-standardized prevalence rate; (e, f) Age-standardized DALYs rate. [file Image1.jpeg]

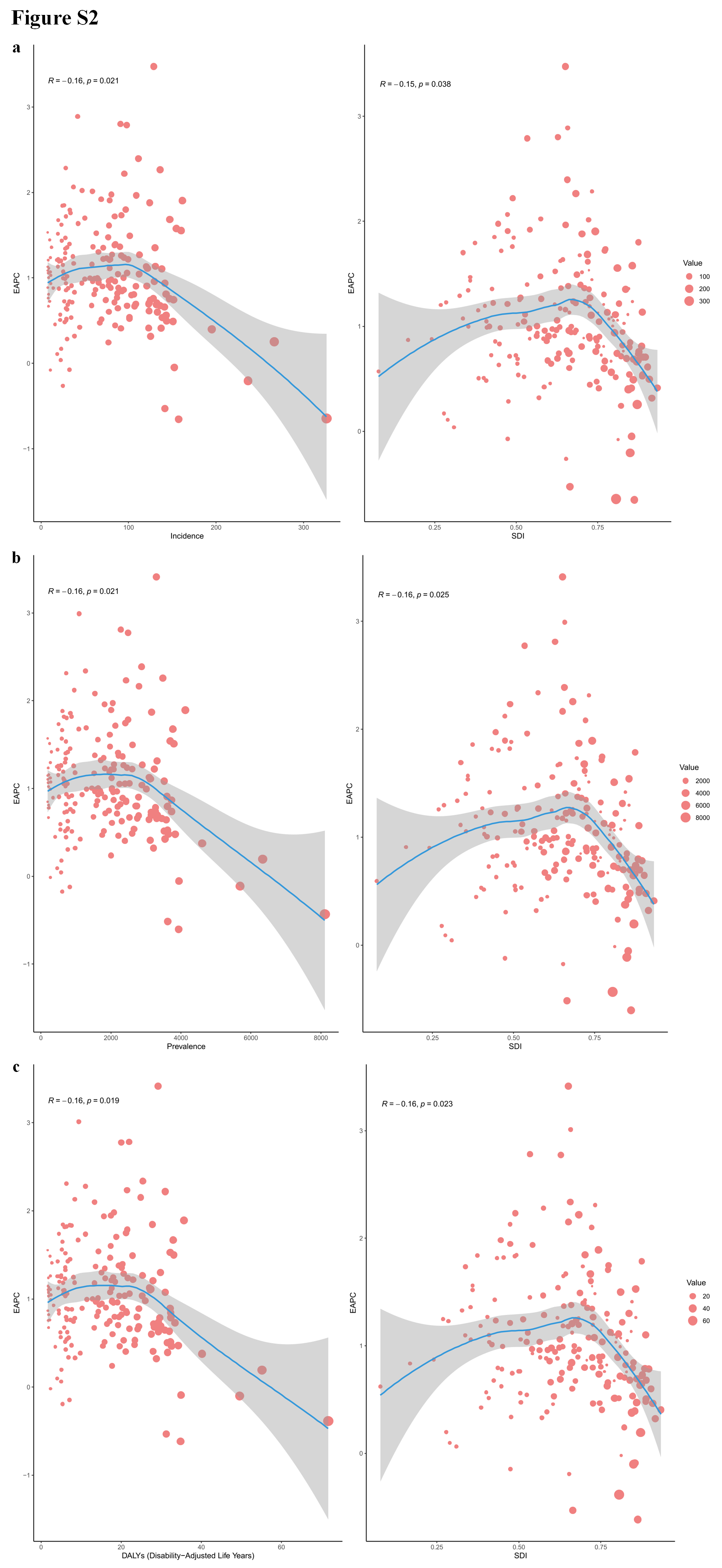

Supplement: Supplementary Figure 2 — Correlation between Estimated Annual Percentage Change (EAPC) in 2021 and age-standardized rates of polycystic ovary syndrome and SDI. In the left figures, circles represent countries, while in the right figures,circles represent countries for which Human Development Index data is available. The size of each circle isproportional to the number of (a) incident cases, (b) prevalent cases, and (c) DALYs. The ρvalues and p-values were obtained from Pearson correlation analysis. [file Image2.jpeg]

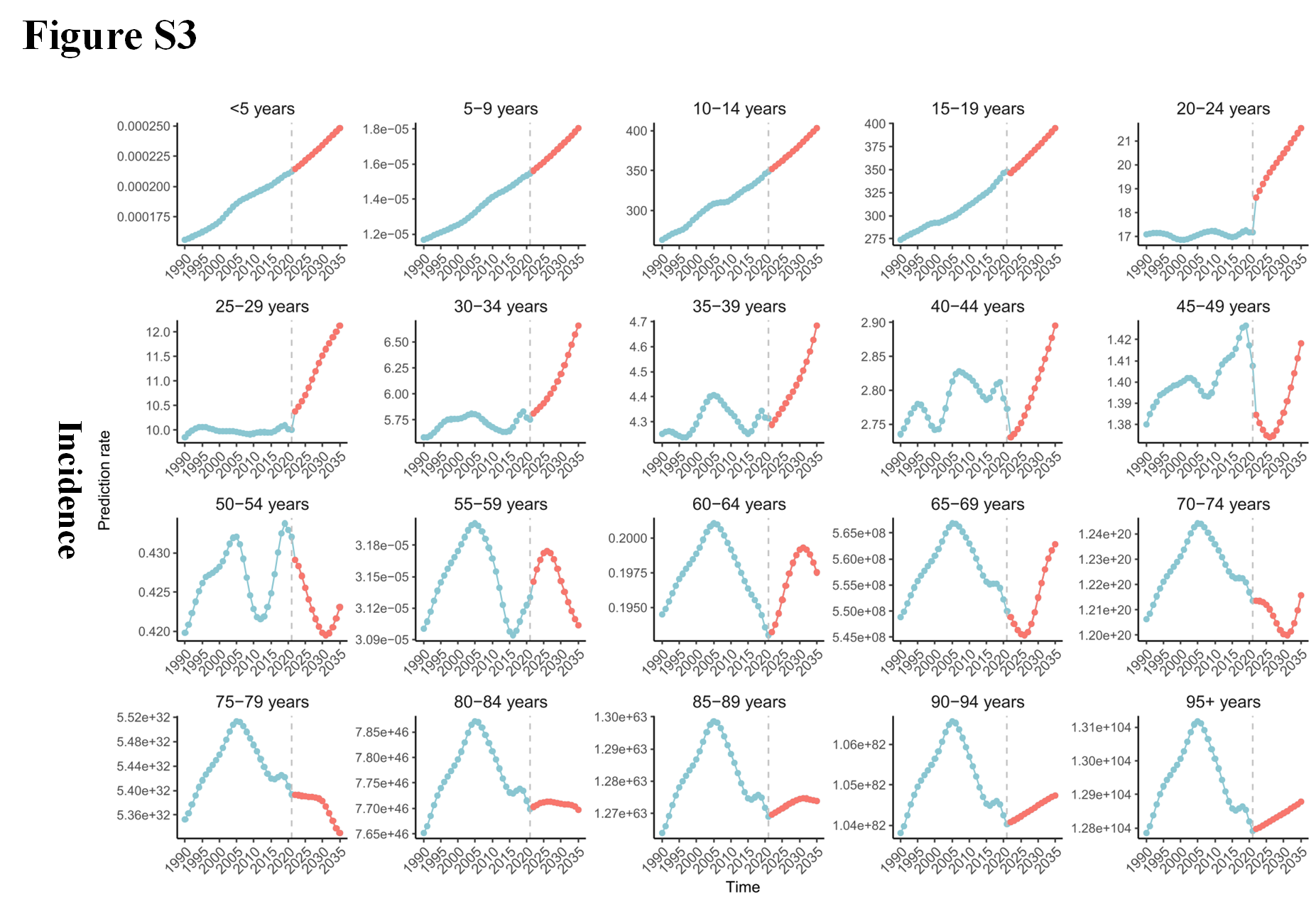

Supplement: Supplementary Figure 3 — Trends of age-standardized incidence rate of polycystic ovary syndrome across age groups: observed rates (1990–2021) and predicted rates (2022–2050). [file Image3.jpeg]

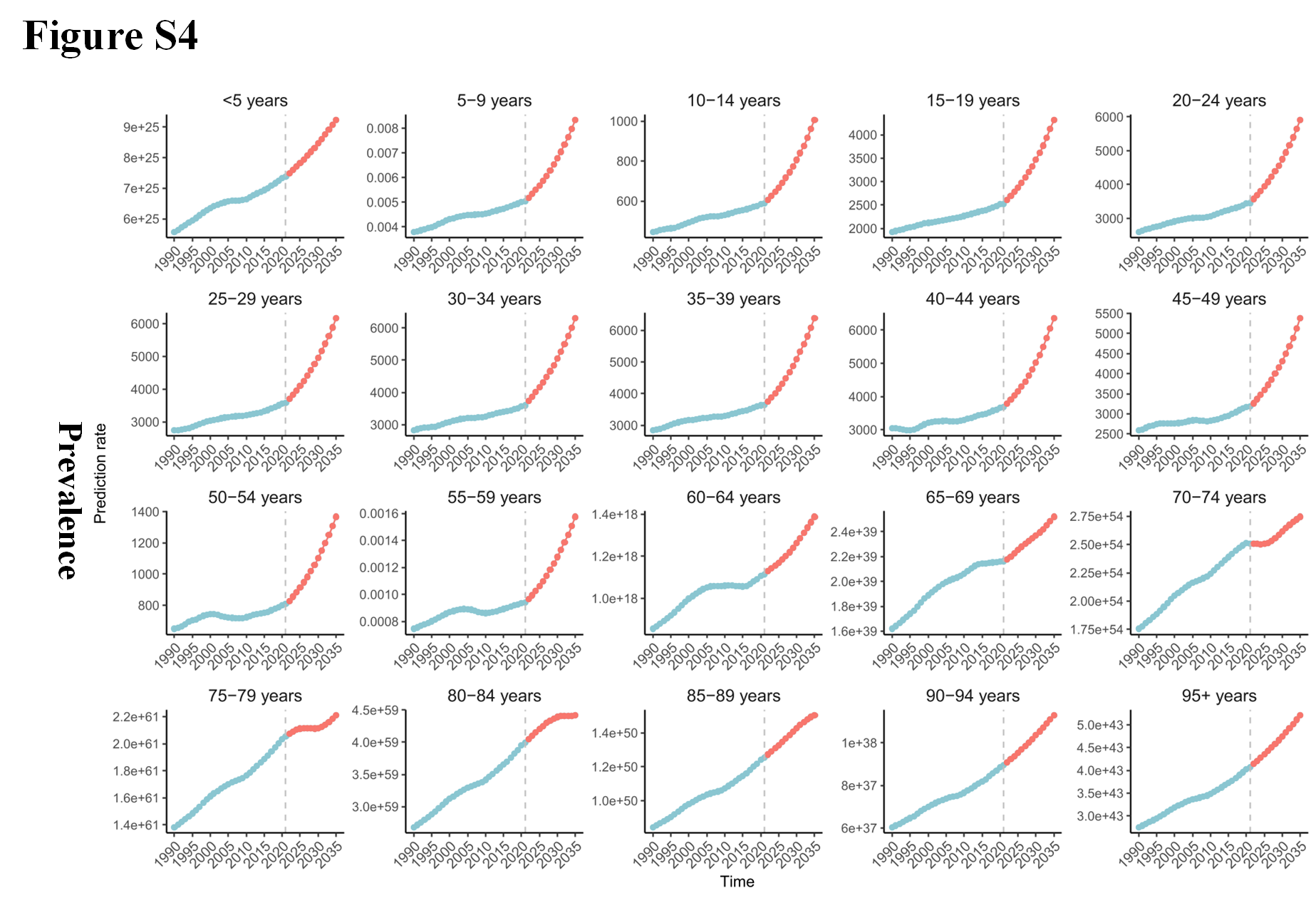

Supplement: Supplementary Figure 4 — Trends of age-standardized prevalence rate of polycystic ovary syndrome across age groups: observed rates (1990–2021) and predicted rates (2022–2050). [file Image4.jpeg]

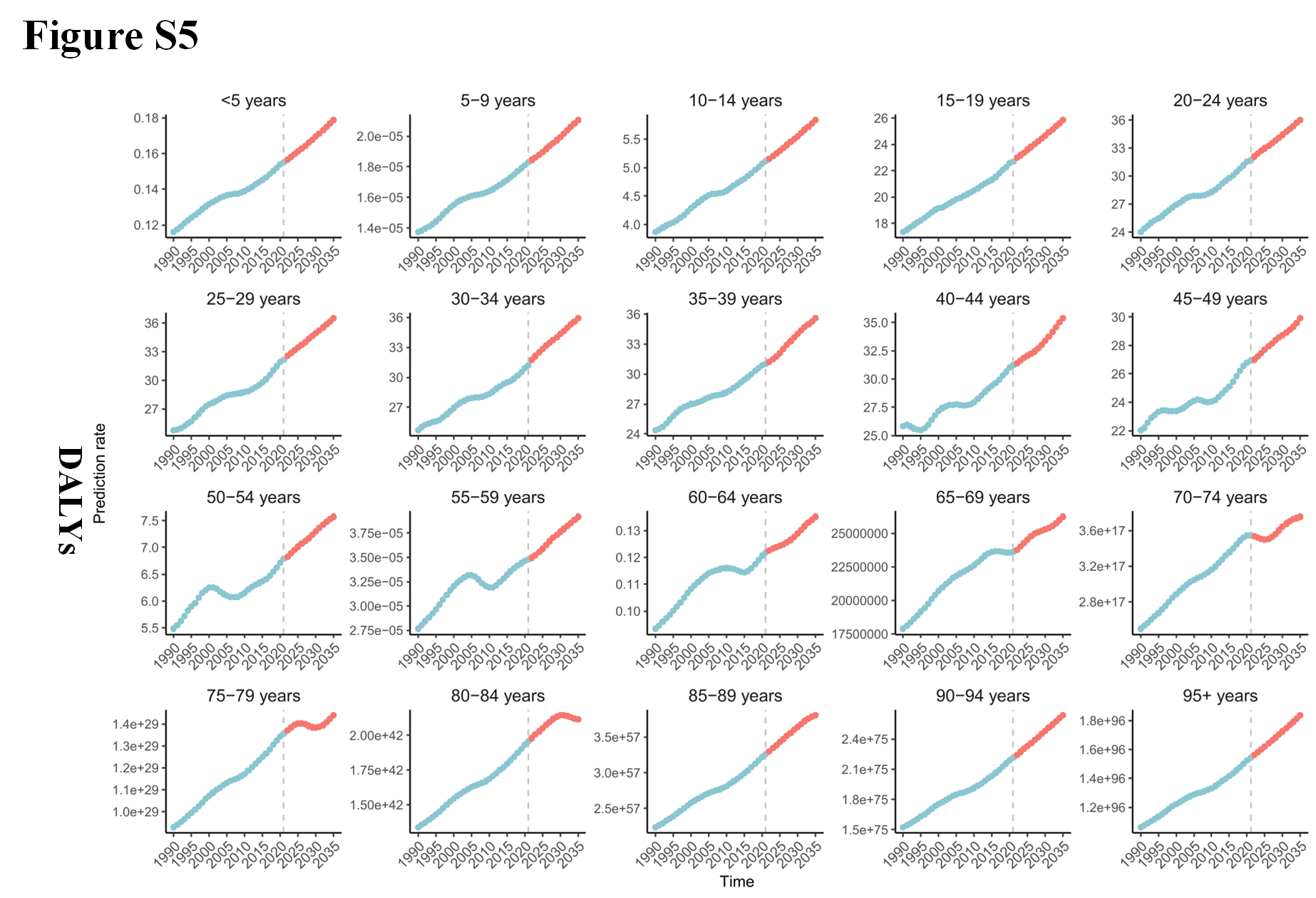

Supplement: Supplementary Figure 5 — Trends of age-standardized DALYs rate of polycystic ovary syndrome across age groups: observed rates (1990–2021) and predicted rates (2022–2050). [file Image5.jpeg]
