# Supplementary material for: Global, regional and national burden of polycystic ovary syndrome: historical trends from 1990 to 2021 and projections to 2035
Source: Front Endocrinol (Lausanne). 2026 Apr 1;17:1662823. doi: 10.3389/fendo.2026.1662823 (PMC13079045; doi:10.3389/fendo.2026.1662823)
Supplement: Supplementary file 6 [file DataSheet1.doc]

**Table S1. The number of incident cases, prevalent cases, and DALYs of polycystic ovary syndrome in 1990, along with corresponding ASR.**

| Characteristics | Incidence(95% uncertainty interval) | |  | Prevalence(95% uncertainty interval) | |  | DALYs(95% uncertainty interval) | |
| --- | --- | --- | --- | --- | --- | --- | --- | --- |
|  | Cases,1990 | ASIR,1990 |  | Cases,1990 | ASPR,1990 |  | Cases,1990 | ASDR,1990 |
| Global | 1476225.27(1057983.50,2045276.94) | 49.45(35.57,68.45) |  | 36651157.24(26227943.17,50603929.78) | 1372.77(984.64,1891.60) |  | 323798.59(144342.15,675926.83) | 12.08(5.38,25.21) |
| SDI level |  |  |  |  |  |  |  |  |
| High SDI | 437301.50(319330.75,606631.95) | 120.81(88.32,167.22) |  | 13783058.46(10021809.44,19223038.27) | 3007.94(2186.43,4172.47) |  | 122087.07(55322.29,254194.96) | 26.66(12.08,55.34) |
| High-middle SDI | 261030.54(187118.65,358760.32) | 49.47(35.43,68.60) |  | 7047030.00(4981606.26,9727416.64) | 1262.73(892.37,1745.92) |  | 61793.58(27718.57,128454.50) | 11.05(4.96,22.99) |
| Middle SDI | 505653.92(359587.99,703114.26) | 48.52(34.54,67.44) |  | 10580143.20(7410905.26,14674450.81) | 1176.94(825.22,1630.44) |  | 93350.16(41377.04,195580.27) | 10.32(4.57,21.76) |
| Low-middle SDI | 209333.49(148000.10,293837.26) | 29.32(21.09,40.77) |  | 4094311.31(2868780.90,5765430.73) | 745.13(526.92,1040.82) |  | 36511.83(15811.37,76977.48) | 6.59(2.84,13.85) |
| Low SDI | 61881.63(43355.56,87726.03) | 20.08(14.45,28.07) |  | 1121930.97(784807.02,1611458.42) | 500.06(353.56,711.57) |  | 9836.52(4213.82,20814.37) | 4.35(1.86,9.22) |
| GBD Region |  |  |  |  |  |  |  |  |
| Andean Latin America | 25250.91(17294.54,36023.12) | 100.30(68.88,142.65) |  | 466365.78(322493.99,653112.67) | 2419.72(1673.59,3379.44) |  | 4055.89(1797.33,8748.39) | 20.98(9.37,45.26) |
| Australasia | 15948.65(11745.49,20802.41) | 173.96(127.23,229.44) |  | 444239.31(326487.36,585953.23) | 4122.50(3027.98,5452.98) |  | 3873.53(1763.71,8060.68) | 35.97(16.34,74.76) |
| Caribbean | 9226.66(6288.13,13042.76) | 44.33(30.33,62.54) |  | 222432.35(149139.10,319229.69) | 1186.23(799.13,1702.73) |  | 1979.83(867.32,4095.22) | 10.50(4.58,21.73) |
| Central Asia | 5347.79(3686.14,7702.43) | 13.68(9.46,19.63) |  | 118817.93(79820.20,174949.99) | 348.50(234.84,511.40) |  | 1049.10(438.14,2236.92) | 3.06(1.28,6.52) |
| Central Europe | 4244.91(2905.82,6223.94) | 7.35(5.01,10.81) |  | 116151.96(76720.98,173921.49) | 185.82(122.77,278.52) |  | 1014.73(425.90,2088.93) | 1.63(0.68,3.36) |
| Central Latin America | 119114.89(81948.23,168844.63) | 107.23(73.94,151.71) |  | 2295521.99(1578370.65,3199568.77) | 2695.62(1856.93,3736.43) |  | 20163.69(9003.77,42043.01) | 23.55(10.46,49.22) |
| Central Sub-Saharan Africa | 6063.01(4247.42,8685.30) | 17.63(12.55,24.87) |  | 110521.94(76168.15,160819.70) | 445.38(309.50,646.83) |  | 958.26(406.25,1975.20) | 3.83(1.63,8.00) |
| East Asia | 218485.79(154343.69,301132.94) | 32.14(22.85,44.87) |  | 5607180.87(3957250.24,7863221.60) | 845.57(600.94,1185.47) |  | 48225.91(20818.56,100142.96) | 7.25(3.13,15.07) |
| Eastern Europe | 8328.34(5913.32,11639.17) | 8.56(5.98,12.09) |  | 237422.15(160523.33,339244.51) | 206.51(139.23,298.06) |  | 2097.69(868.62,4466.82) | 1.83(0.76,3.89) |
| Eastern Sub-Saharan Africa | 25901.96(18214.02,36986.15) | 20.71(14.82,29.05) |  | 448071.03(310256.93,646236.16) | 516.67(364.08,741.26) |  | 3903.29(1645.09,8198.57) | 4.46(1.88,9.40) |
| High-income Asia Pacific | 158931.18(111210.64,226424.22) | 193.02(137.42,268.99) |  | 4402355.40(3172199.05,6106156.59) | 4750.21(3430.32,6586.66) |  | 38274.50(16930.42,77128.81) | 41.40(18.34,83.40) |
| High-income North America | 134112.71(94940.50,187144.22) | 119.24(83.99,166.82) |  | 4469583.70(3157929.56,6274672.88) | 2975.32(2098.69,4185.41) |  | 39976.10(17688.81,82638.29) | 26.60(11.77,54.70) |
| North Africa and Middle East | 128593.52(88937.33,183308.60) | 59.01(41.15,83.29) |  | 2463301.08(1707181.32,3501813.36) | 1548.77(1073.96,2200.34) |  | 22386.47(9812.91,47102.56) | 13.96(6.10,29.29) |
| Oceania | 2013.15(1401.24,2847.04) | 49.56(34.79,69.76) |  | 40226.69(27505.04,56891.57) | 1278.85(881.22,1798.41) |  | 352.20(160.65,738.87) | 11.14(5.06,23.30) |
| South Asia | 162363.29(116293.11,226382.69) | 25.33(18.35,34.97) |  | 3294301.20(2344772.01,4593824.79) | 642.80(462.57,893.35) |  | 29342.13(12824.11,62333.75) | 5.68(2.49,12.07) |
| Southeast Asia | 174333.20(122499.01,243431.39) | 58.95(41.50,82.07) |  | 3682948.82(2589198.76,5201509.94) | 1522.31(1075.34,2154.40) |  | 32853.77(14429.10,66791.76) | 13.50(5.92,27.49) |
| Southern Latin America | 12429.77(8602.22,17727.21) | 46.78(32.37,66.78) |  | 296321.47(204840.66,431020.07) | 1185.87(822.02,1727.03) |  | 2627.83(1179.43,5421.35) | 10.50(4.71,21.66) |
| Southern Sub-Saharan Africa | 11799.72(8261.02,16787.82) | 33.79(23.76,47.64) |  | 232808.63(160575.02,334631.38) | 870.65(601.45,1246.58) |  | 2047.42(881.84,4435.18) | 7.59(3.27,16.39) |
| Tropical Latin America | 22284.58(15199.83,32003.99) | 23.31(15.97,33.28) |  | 448844.15(304409.01,647659.16) | 558.68(379.28,801.65) |  | 4011.07(1701.11,8427.30) | 4.97(2.11,10.47) |
| Western Europe | 206740.90(146330.87,286172.09) | 143.35(100.92,200.55) |  | 6815142.46(4796290.79,9455180.18) | 3547.26(2498.52,4925.03) |  | 60788.92(27584.87,126652.56) | 31.64(14.36,65.86) |
| Western Sub-Saharan Africa | 24710.33(17219.71,35159.50) | 20.04(14.40,27.97) |  | 438598.33(307139.67,633877.78) | 501.95(354.54,717.07) |  | 3816.23(1628.96,8015.42) | 4.32(1.85,9.10) |
