# Supplementary material for: Global, regional and national burden of polycystic ovary syndrome: historical trends from 1990 to 2021 and projections to 2035
Source: Front Endocrinol (Lausanne). 2026 Apr 1;17:1662823. doi: 10.3389/fendo.2026.1662823 (PMC13079045; doi:10.3389/fendo.2026.1662823)
Supplement: Supplementary file 7 [file DataSheet2.doc]

**Table S2. The number of incident cases, prevalent cases, and DALYs of polycystic ovary syndrome at the National Level in 2021, along with corresponding ASR and EAPC.**

| Characteristics | Incidence(95% uncertainty interval) | | |  | Prevalence(95% uncertainty interval) | | |  | DALYs(95% uncertainty interval) | | |
| --- | --- | --- | --- | --- | --- | --- | --- | --- | --- | --- | --- |
|  | Cases,2021 | ASIR,2021 | EAPC 1990-2021 |  | Cases,2021 | ASPR,2021 | EAPC 1990-2021 |  | Cases,2021 | ASDR,2021 | EAPC 1990-2021 |
| Afghanistan | 10509.12(7174.58,15049.51) | 50.11(34.40,71.40) | 1.70(1.41,1.99) |  | 189341.71(128908.62,270540.45) | 1295.31(889.64,1847.76) | 1.69(1.40,1.98) |  | 1640.04(706.46,3495.41) | 11.11(4.78,23.89) | 1.73(1.44,2.03) |
| Albania | 71.90(49.57,104.15) | 7.70(5.23,11.29) | 0.89(0.79,0.98) |  | 2384.01(1587.36,3625.07) | 191.44(126.23,291.31) | 0.85(0.75,0.94) |  | 21.07(8.93,45.76) | 1.69(0.72,3.69) | 0.87(0.76,0.97) |
| Algeria | 17007.74(11426.08,24406.49) | 84.01(56.67,120.59) | 1.38(1.34,1.43) |  | 500093.01(338802.22,717341.10) | 2205.99(1497.75,3172.01) | 1.41(1.36,1.45) |  | 4388.99(1907.40,9207.83) | 19.42(8.47,40.73) | 1.38(1.32,1.44) |
| American Samoa | 31.37(21.74,45.85) | 108.31(75.38,157.79) | 0.90(0.72,1.09) |  | 662.34(463.12,968.64) | 2789.56(1942.90,4075.00) | 0.90(0.72,1.09) |  | 5.78(2.51,12.21) | 24.41(10.56,51.54) | 0.89(0.71,1.07) |
| Andorra | 33.44(23.11,47.10) | 140.95(97.02,199.68) | 0.66(0.55,0.77) |  | 1444.29(1002.83,2030.62) | 3580.57(2469.01,5055.23) | 0.64(0.54,0.75) |  | 12.76(5.67,26.69) | 32.02(14.34,67.46) | 0.63(0.53,0.74) |
| Angola | 6341.64(4314.27,9185.27) | 28.41(19.49,40.98) | 1.72(1.58,1.86) |  | 114150.30(77525.53,165941.41) | 734.31(499.67,1062.30) | 1.80(1.66,1.95) |  | 993.38(423.17,2020.46) | 6.34(2.72,12.95) | 1.82(1.68,1.97) |
| Antigua and Barbuda | 21.71(14.98,30.61) | 60.64(41.80,86.38) | 0.81(0.75,0.86) |  | 765.37(525.47,1089.18) | 1577.69(1081.35,2242.53) | 0.81(0.75,0.87) |  | 6.72(2.97,13.72) | 13.92(6.13,28.54) | 0.80(0.75,0.86) |
| Argentina | 14464.77(10257.15,20683.25) | 70.92(50.34,101.64) | 1.35(1.18,1.53) |  | 430891.69(302555.84,613693.23) | 1794.47(1259.48,2552.32) | 1.35(1.17,1.52) |  | 3809.96(1682.55,7869.12) | 15.89(7.02,32.73) | 1.35(1.17,1.52) |
| Armenia | 199.49(137.00,283.64) | 19.23(13.21,27.95) | 1.52(1.45,1.58) |  | 7142.76(4825.97,10368.32) | 478.73(323.90,700.39) | 1.44(1.39,1.50) |  | 61.64(26.56,130.93) | 4.15(1.79,8.78) | 1.43(1.37,1.48) |
| Australia | 17110.95(12186.75,23869.24) | 195.10(138.82,272.46) | 0.40(0.27,0.53) |  | 563726.99(398303.52,791359.88) | 4606.14(3265.61,6417.26) | 0.38(0.25,0.50) |  | 4904.34(2199.30,10306.43) | 40.20(18.03,83.48) | 0.38(0.25,0.50) |
| Austria | 3758.21(2632.31,5235.34) | 152.33(105.34,213.46) | -0.05(-0.13,0.03) |  | 157940.02(109288.81,222554.32) | 3942.67(2723.35,5569.98) | -0.05(-0.13,0.02) |  | 1390.53(608.77,2855.12) | 34.94(15.26,71.48) | -0.09(-0.18,-0.00) |
| Azerbaijan | 818.45(559.91,1129.53) | 19.73(13.21,27.45) | 1.68(1.56,1.79) |  | 28152.08(18861.19,40757.90) | 505.58(337.65,727.07) | 1.73(1.61,1.85) |  | 246.87(101.12,537.92) | 4.46(1.81,9.72) | 1.74(1.61,1.86) |
| Bahamas | 135.89(92.34,198.23) | 69.02(46.90,100.80) | 0.52(0.45,0.59) |  | 3896.61(2635.44,5665.69) | 1796.01(1213.40,2608.97) | 0.52(0.44,0.59) |  | 34.33(15.23,73.50) | 15.86(7.03,33.97) | 0.51(0.44,0.58) |
| Bahrain | 499.93(350.45,703.67) | 91.28(63.71,129.23) | 0.41(0.36,0.46) |  | 15710.06(10800.87,22287.73) | 2399.15(1652.22,3399.21) | 0.40(0.35,0.45) |  | 138.06(60.68,291.91) | 21.12(9.30,44.61) | 0.40(0.35,0.45) |
| Bangladesh | 22460.10(15709.10,31143.09) | 24.43(17.17,33.89) | 1.84(1.70,1.99) |  | 569396.86(391140.02,811981.31) | 614.52(422.62,875.78) | 1.88(1.74,2.02) |  | 5038.24(2153.38,10681.52) | 5.43(2.32,11.50) | 1.84(1.71,1.97) |
| Barbados | 70.76(48.60,99.19) | 67.19(45.35,94.29) | 0.47(0.44,0.51) |  | 2498.88(1711.30,3562.21) | 1747.68(1187.58,2498.66) | 0.47(0.44,0.50) |  | 21.92(9.63,46.86) | 15.41(6.75,33.00) | 0.47(0.43,0.50) |
| Belarus | 310.29(218.36,439.84) | 10.64(7.38,15.35) | 1.07(0.99,1.15) |  | 11603.28(7955.82,16946.94) | 265.96(180.29,396.60) | 1.12(1.04,1.20) |  | 101.18(42.09,220.14) | 2.35(0.98,5.15) | 1.11(1.02,1.19) |
| Belgium | 5059.84(3571.52,7107.14) | 137.03(96.60,192.74) | 0.41(0.21,0.61) |  | 178151.13(124278.17,248708.98) | 3545.39(2465.51,4947.93) | 0.42(0.23,0.62) |  | 1577.15(720.57,3300.16) | 31.59(14.47,66.14) | 0.39(0.20,0.58) |
| Belize | 169.41(116.92,241.56) | 65.92(44.95,94.84) | 0.97(0.71,1.24) |  | 4130.05(2832.09,5831.06) | 1698.95(1166.35,2399.27) | 0.97(0.70,1.24) |  | 36.90(16.26,78.74) | 15.12(6.67,32.25) | 0.94(0.68,1.21) |
| Benin | 2958.36(1989.24,4268.45) | 32.87(22.40,46.94) | 1.79(1.53,2.05) |  | 55218.49(37120.55,79494.68) | 841.31(570.30,1209.54) | 1.86(1.58,2.14) |  | 476.46(205.49,1010.64) | 7.20(3.11,15.23) | 1.84(1.57,2.11) |
| Bermuda | 13.63(9.39,19.99) | 77.01(52.53,113.93) | 0.24(0.18,0.30) |  | 542.79(373.35,787.34) | 2003.91(1376.50,2920.75) | 0.24(0.18,0.30) |  | 4.75(2.03,9.91) | 17.71(7.61,36.75) | 0.24(0.18,0.30) |
| Bhutan | 142.58(99.13,202.45) | 36.96(25.68,52.75) | 2.06(1.94,2.19) |  | 3965.30(2711.67,5692.35) | 951.35(651.15,1365.52) | 2.12(1.99,2.25) |  | 35.09(15.36,73.82) | 8.41(3.68,17.67) | 2.13(2.00,2.26) |
| Bolivia (Plurinational State of) | 7483.00(5046.17,10512.30) | 120.85(81.51,169.90) | 0.91(0.87,0.95) |  | 189605.02(127099.58,268471.94) | 3013.26(2024.42,4266.89) | 0.99(0.92,1.07) |  | 1638.17(713.68,3514.50) | 26.01(11.34,55.81) | 0.98(0.90,1.05) |
| Bosnia and Herzegovina | 77.74(52.75,112.63) | 7.59(5.07,11.17) | 1.53(1.32,1.75) |  | 2841.85(1865.97,4326.01) | 191.47(125.22,293.78) | 1.57(1.35,1.79) |  | 24.62(10.40,53.00) | 1.67(0.70,3.64) | 1.55(1.33,1.77) |
| Botswana | 544.52(372.16,764.25) | 42.27(28.68,59.53) | 1.64(1.43,1.84) |  | 14821.67(10044.27,20992.47) | 1081.33(733.16,1530.54) | 1.68(1.46,1.89) |  | 126.37(55.05,265.04) | 9.23(4.02,19.29) | 1.67(1.46,1.88) |
| Brazil | 22886.08(16023.83,31692.70) | 24.97(17.19,35.09) | -0.26(-0.45,-0.08) |  | 723408.08(499293.64,1022776.74) | 610.20(419.76,868.28) | -0.17(-0.35,-0.00) |  | 6354.86(2741.03,13399.72) | 5.38(2.33,11.29) | -0.19(-0.37,-0.02) |
| Brunei Darussalam | 298.43(205.92,421.57) | 160.01(111.25,224.46) | 1.55(1.39,1.72) |  | 9654.38(6789.03,13508.21) | 3797.30(2681.33,5308.98) | 1.51(1.35,1.67) |  | 84.09(38.07,172.43) | 33.12(15.02,68.00) | 1.50(1.34,1.66) |
| Bulgaria | 167.92(116.07,244.91) | 8.65(5.87,12.80) | 0.81(0.77,0.84) |  | 6314.95(4208.51,9406.47) | 218.84(144.56,326.54) | 0.81(0.77,0.85) |  | 54.35(22.57,113.43) | 1.90(0.77,3.97) | 0.82(0.78,0.86) |
| Burkina Faso | 4149.60(2845.64,5824.99) | 27.10(18.90,37.99) | 1.23(1.03,1.42) |  | 76846.27(52612.38,110149.78) | 691.63(474.65,990.30) | 1.30(1.09,1.51) |  | 669.31(283.37,1426.45) | 5.98(2.53,12.65) | 1.23(1.03,1.43) |
| Burundi | 1664.01(1145.37,2344.12) | 18.62(13.00,25.75) | 0.11(0.04,0.18) |  | 28909.32(19638.51,41714.41) | 460.99(314.65,664.66) | 0.09(0.01,0.17) |  | 251.12(104.84,525.19) | 3.98(1.68,8.30) | 0.10(0.00,0.19) |
| Cabo Verde | 94.00(65.78,133.54) | 32.43(22.54,45.90) | 1.40(1.19,1.61) |  | 2540.79(1745.75,3680.76) | 837.21(575.08,1210.69) | 1.45(1.23,1.67) |  | 21.92(9.48,45.96) | 7.22(3.12,15.10) | 1.45(1.23,1.67) |
| Cambodia | 7003.85(4910.38,9987.18) | 78.36(54.92,111.79) | 1.91(1.86,1.95) |  | 183686.37(127720.29,263849.33) | 2006.79(1398.68,2881.22) | 1.89(1.86,1.93) |  | 1597.13(708.30,3275.46) | 17.44(7.75,35.71) | 1.95(1.90,1.99) |
| Cameroon | 7517.97(4966.64,10838.83) | 35.51(23.66,50.89) | 0.71(0.62,0.81) |  | 146559.59(97348.72,216294.12) | 922.85(613.78,1361.89) | 0.74(0.64,0.84) |  | 1294.97(554.10,2720.99) | 8.08(3.48,17.05) | 0.74(0.64,0.85) |
| Canada | 8840.85(6149.48,12717.30) | 73.82(51.22,106.18) | 0.74(0.63,0.84) |  | 310637.33(217280.08,450465.33) | 1841.49(1289.91,2661.42) | 0.72(0.62,0.82) |  | 2769.44(1177.80,5631.64) | 16.48(7.06,33.24) | 0.71(0.61,0.82) |
| Central African Republic | 824.26(567.95,1166.14) | 22.41(15.60,31.42) | 0.04(-0.08,0.16) |  | 15978.90(10841.54,23053.97) | 575.39(392.54,824.48) | 0.04(-0.08,0.17) |  | 140.56(60.18,299.46) | 5.02(2.15,10.67) | 0.06(-0.06,0.19) |
| Chad | 2462.78(1683.69,3519.57) | 20.44(14.31,28.68) | 0.88(0.66,1.10) |  | 40291.35(27733.96,58316.95) | 517.40(356.47,746.80) | 0.90(0.66,1.15) |  | 346.89(148.27,742.52) | 4.41(1.91,9.39) | 0.87(0.61,1.13) |
| Chile | 6057.22(4275.77,8709.62) | 84.21(59.19,121.13) | 1.72(1.40,2.04) |  | 203583.43(142311.75,295879.54) | 2126.29(1491.17,3082.69) | 1.72(1.40,2.03) |  | 1791.92(763.82,3675.71) | 18.75(8.02,38.32) | 1.70(1.39,2.02) |
| China | 258930.01(183411.57,361501.06) | 58.61(41.19,82.36) | 2.01(1.86,2.17) |  | 10077520.01(7120017.13,14249277.28) | 1544.17(1081.83,2169.82) | 2.08(1.92,2.25) |  | 86442.99(37911.10,178421.14) | 13.34(5.88,27.55) | 2.10(1.93,2.27) |
| Colombia | 18351.36(12636.42,26802.64) | 86.70(59.59,127.64) | 0.77(0.71,0.83) |  | 578236.93(394399.95,838014.81) | 2190.96(1493.43,3183.33) | 0.79(0.73,0.85) |  | 5136.56(2219.59,10918.16) | 19.45(8.41,41.39) | 0.79(0.74,0.85) |
| Comoros | 129.41(88.82,182.99) | 29.89(20.52,42.21) | 0.28(0.12,0.45) |  | 3037.44(2080.89,4386.12) | 771.62(529.54,1112.21) | 0.31(0.14,0.47) |  | 27.10(11.70,56.10) | 6.86(2.96,14.20) | 0.34(0.17,0.50) |
| Congo | 1055.67(725.58,1508.25) | 30.79(21.28,43.98) | 0.88(0.75,1.01) |  | 22946.66(15866.20,32882.52) | 794.25(548.10,1139.69) | 0.90(0.77,1.04) |  | 196.55(86.86,415.56) | 6.79(2.99,14.29) | 0.88(0.72,1.04) |
| Cook Islands | 9.78(6.79,14.15) | 120.25(82.98,174.65) | 1.13(0.99,1.27) |  | 268.96(183.71,390.80) | 3094.71(2112.97,4501.41) | 1.13(0.99,1.27) |  | 2.35(1.04,5.04) | 27.02(11.92,58.02) | 1.13(0.99,1.26) |
| Costa Rica | 2155.43(1461.82,3075.02) | 106.18(71.68,151.78) | 0.81(0.74,0.87) |  | 71476.92(48426.25,103396.50) | 2742.27(1860.44,3961.65) | 0.80(0.74,0.86) |  | 623.46(269.19,1351.39) | 23.96(10.36,52.06) | 0.80(0.73,0.86) |
| Côte d'Ivoire | 5019.40(3426.24,7345.24) | 29.82(20.56,43.32) | 1.36(1.08,1.63) |  | 103692.98(71072.51,152858.84) | 769.68(528.59,1131.62) | 1.42(1.13,1.71) |  | 898.38(391.63,1901.47) | 6.63(2.89,14.01) | 1.43(1.14,1.72) |
| Croatia | 104.74(72.20,149.77) | 8.37(5.60,12.16) | 1.05(0.95,1.16) |  | 3861.74(2549.98,5789.97) | 211.96(139.84,319.03) | 1.07(0.97,1.17) |  | 33.55(13.49,71.41) | 1.85(0.75,3.94) | 1.08(0.98,1.18) |
| Cuba | 2304.09(1570.71,3214.97) | 66.06(44.66,92.86) | 0.94(0.89,0.99) |  | 86670.29(58800.05,122862.28) | 1720.78(1167.50,2445.64) | 0.94(0.90,0.99) |  | 759.94(328.86,1594.31) | 15.18(6.57,31.88) | 0.95(0.90,1.00) |
| Cyprus | 500.45(347.14,715.96) | 130.16(89.94,187.01) | 1.35(1.17,1.53) |  | 23926.08(16536.18,34357.57) | 3316.40(2294.61,4779.62) | 1.31(1.14,1.48) |  | 213.39(94.82,437.47) | 29.81(13.38,61.05) | 1.30(1.13,1.47) |
| Czechia | 261.58(179.27,382.88) | 8.17(5.52,12.08) | 0.76(0.71,0.82) |  | 9601.21(6367.50,14247.00) | 207.67(136.98,311.84) | 0.78(0.72,0.84) |  | 82.67(34.83,176.66) | 1.80(0.75,3.78) | 0.76(0.70,0.82) |
| Democratic People's Republic of Korea | 3897.38(2717.14,5421.25) | 38.25(26.36,53.31) | 0.32(0.23,0.41) |  | 131299.53(90384.86,184878.03) | 980.60(674.56,1378.28) | 0.32(0.23,0.41) |  | 1132.02(485.49,2360.27) | 8.48(3.64,17.74) | 0.33(0.24,0.42) |
| Democratic Republic of the Congo | 14822.35(10269.82,21198.89) | 24.89(17.44,35.27) | 1.15(0.99,1.32) |  | 273378.97(187214.58,395201.31) | 635.93(437.90,918.11) | 1.19(1.03,1.36) |  | 2378.49(1034.15,4884.56) | 5.49(2.38,11.37) | 1.18(1.00,1.37) |
| Denmark | 2516.88(1744.53,3582.05) | 132.29(90.98,188.09) | 0.71(0.60,0.81) |  | 83546.78(58299.98,115406.11) | 3274.15(2274.55,4525.94) | 0.78(0.65,0.91) |  | 753.45(344.23,1540.92) | 29.59(13.50,60.47) | 0.79(0.66,0.91) |
| Djibouti | 212.39(144.10,308.85) | 33.73(22.91,49.09) | 1.76(1.63,1.89) |  | 5668.98(3824.64,8246.86) | 875.22(590.56,1273.90) | 1.82(1.68,1.96) |  | 49.05(21.48,105.04) | 7.58(3.33,16.22) | 1.83(1.69,1.97) |
| Dominica | 19.53(13.17,27.53) | 62.11(41.74,88.32) | 0.86(0.74,0.98) |  | 537.68(362.12,773.61) | 1614.82(1087.50,2326.90) | 0.86(0.74,0.98) |  | 4.75(2.08,9.99) | 14.28(6.26,30.00) | 0.85(0.73,0.98) |
| Dominican Republic | 3377.77(2268.23,4794.42) | 63.06(42.23,90.00) | 1.52(1.43,1.62) |  | 95011.02(64011.85,139093.85) | 1628.37(1096.59,2382.98) | 1.56(1.46,1.65) |  | 835.60(354.58,1732.25) | 14.30(6.06,29.63) | 1.54(1.44,1.64) |
| Ecuador | 14008.33(9802.69,19335.75) | 151.32(105.93,208.83) | 0.74(0.46,1.03) |  | 355533.05(250363.64,492188.63) | 3731.44(2625.38,5158.52) | 0.87(0.63,1.11) |  | 3085.86(1353.85,6419.97) | 32.36(14.20,67.26) | 0.84(0.60,1.08) |
| Egypt | 50586.97(35062.59,71899.99) | 87.73(61.35,124.82) | 0.64(0.58,0.71) |  | 1213934.66(839684.14,1721360.76) | 2307.93(1600.55,3266.64) | 0.63(0.56,0.70) |  | 10871.03(4840.84,22507.29) | 20.62(9.17,42.88) | 0.53(0.46,0.60) |
| El Salvador | 3062.85(2061.61,4439.64) | 94.25(63.08,136.91) | 1.00(0.86,1.14) |  | 86019.12(58999.66,123358.60) | 2401.53(1646.79,3446.62) | 1.06(0.95,1.16) |  | 749.46(327.65,1572.97) | 20.90(9.13,43.82) | 1.04(0.93,1.14) |
| Equatorial Guinea | 385.01(262.40,546.84) | 41.85(28.77,59.15) | 2.89(2.46,3.32) |  | 8049.14(5441.04,11571.33) | 1093.30(742.52,1576.30) | 2.99(2.54,3.44) |  | 69.74(30.60,143.12) | 9.41(4.12,19.17) | 3.01(2.56,3.47) |
| Eritrea | 902.65(632.80,1300.31) | 22.62(15.90,32.39) | 1.19(1.00,1.39) |  | 19234.90(13121.78,28015.83) | 577.34(393.92,836.34) | 1.27(1.06,1.47) |  | 168.65(69.37,357.19) | 5.04(2.10,10.68) | 1.27(1.07,1.46) |
| Estonia | 49.25(34.47,71.51) | 11.98(8.26,17.46) | 1.36(1.27,1.45) |  | 1719.63(1156.57,2548.76) | 301.92(202.68,453.96) | 1.42(1.33,1.51) |  | 15.10(6.23,33.36) | 2.68(1.11,5.90) | 1.41(1.32,1.50) |
| Eswatini | 315.48(217.47,444.28) | 44.09(30.37,62.02) | 0.42(0.16,0.68) |  | 7165.24(4897.30,10138.74) | 1123.36(768.00,1598.30) | 0.42(0.17,0.68) |  | 62.69(26.43,127.49) | 9.78(4.11,19.83) | 0.38(0.13,0.64) |
| Ethiopia | 17269.79(12201.09,24551.19) | 23.60(16.74,33.05) | 1.44(1.37,1.50) |  | 328062.49(230109.39,477938.89) | 595.16(421.96,860.31) | 1.57(1.50,1.64) |  | 2879.25(1231.79,6264.11) | 5.18(2.21,11.22) | 1.56(1.50,1.62) |
| Fiji | 447.11(310.54,643.11) | 94.66(65.78,135.60) | 1.24(1.09,1.38) |  | 11366.13(7891.69,16307.84) | 2456.86(1705.88,3525.51) | 1.24(1.09,1.38) |  | 100.08(44.35,204.36) | 21.64(9.60,44.18) | 1.24(1.09,1.38) |
| Finland | 2285.25(1567.72,3255.80) | 131.64(90.39,188.24) | 0.69(0.64,0.73) |  | 76672.03(52775.58,107497.63) | 3328.88(2300.34,4678.11) | 0.70(0.65,0.74) |  | 684.90(310.18,1407.37) | 29.88(13.55,61.35) | 0.69(0.65,0.74) |
| France | 28748.84(19847.99,40731.73) | 122.95(84.55,174.44) | 0.69(0.65,0.73) |  | 911482.68(624226.98,1291203.08) | 3174.80(2171.54,4513.88) | 0.70(0.66,0.74) |  | 8230.32(3734.80,17025.95) | 28.86(13.07,59.63) | 0.68(0.64,0.72) |
| Gabon | 443.02(299.98,646.06) | 37.20(25.29,54.15) | 1.17(1.03,1.31) |  | 9616.82(6377.94,14158.85) | 972.75(645.02,1427.42) | 1.18(1.04,1.33) |  | 83.14(35.33,175.71) | 8.36(3.54,17.77) | 1.18(1.04,1.33) |
| Gambia | 455.41(318.09,649.14) | 27.37(19.17,38.79) | 0.99(0.83,1.15) |  | 8704.04(5983.64,12694.21) | 702.01(485.66,1015.59) | 1.03(0.87,1.19) |  | 75.67(32.12,151.60) | 6.05(2.56,12.22) | 1.03(0.87,1.20) |
| Georgia | 332.59(231.51,458.71) | 28.18(19.41,38.83) | 2.29(2.04,2.53) |  | 11496.33(7893.43,15984.42) | 723.25(493.76,1013.69) | 2.31(2.07,2.56) |  | 100.03(43.38,207.98) | 6.34(2.74,13.20) | 2.31(2.06,2.56) |
| Germany | 27013.95(19008.18,37906.16) | 123.43(85.89,173.38) | 0.61(0.57,0.66) |  | 1072458.75(750548.01,1495114.71) | 3088.44(2154.31,4291.35) | 0.65(0.59,0.70) |  | 9512.36(4369.28,19280.66) | 27.54(12.63,55.81) | 0.60(0.55,0.65) |
| Ghana | 5895.02(4176.22,8487.40) | 28.40(20.22,40.84) | 0.94(0.70,1.18) |  | 134389.15(92881.87,196019.46) | 728.65(505.44,1058.76) | 0.93(0.68,1.19) |  | 1159.89(499.16,2360.82) | 6.26(2.70,12.80) | 0.89(0.64,1.14) |
| Greece | 4181.62(2825.08,5959.15) | 142.85(96.28,203.73) | 0.56(0.38,0.75) |  | 158477.76(109353.45,226715.45) | 3616.57(2488.92,5139.19) | 0.52(0.34,0.71) |  | 1400.42(631.49,2948.84) | 32.31(14.56,68.03) | 0.51(0.34,0.69) |
| Greenland | 13.93(9.82,19.97) | 65.26(46.18,93.26) | 0.95(0.86,1.03) |  | 426.27(298.28,604.73) | 1629.81(1146.26,2310.59) | 0.93(0.84,1.02) |  | 3.81(1.71,8.14) | 14.55(6.51,31.04) | 0.93(0.84,1.02) |
| Grenada | 24.76(16.79,34.58) | 55.96(37.72,78.63) | 1.00(0.87,1.13) |  | 752.83(504.49,1071.67) | 1453.75(974.11,2070.27) | 1.00(0.87,1.14) |  | 6.66(2.99,14.28) | 12.85(5.78,27.57) | 1.00(0.87,1.13) |
| Guam | 82.33(57.01,115.80) | 121.65(83.72,171.37) | 1.11(1.02,1.20) |  | 2285.73(1572.91,3261.90) | 3133.82(2156.98,4475.70) | 1.11(1.02,1.20) |  | 20.01(8.85,41.79) | 27.47(12.13,57.31) | 1.11(1.02,1.20) |
| Guatemala | 7581.01(5106.24,10999.82) | 80.20(53.70,116.27) | 0.98(0.87,1.09) |  | 183158.89(120863.16,267188.38) | 2065.87(1361.35,3003.97) | 0.96(0.84,1.08) |  | 1574.00(679.52,3249.67) | 17.70(7.62,36.65) | 0.97(0.85,1.09) |
| Guinea | 2274.48(1585.68,3258.08) | 25.26(17.73,35.88) | 1.07(0.94,1.21) |  | 43311.15(29735.93,62037.01) | 644.69(446.28,924.09) | 1.11(0.97,1.26) |  | 373.49(160.93,810.37) | 5.53(2.38,11.99) | 1.10(0.95,1.25) |
| Guinea-Bissau | 344.45(238.23,495.29) | 25.19(17.47,35.60) | 1.00(0.74,1.26) |  | 6832.07(4700.85,9925.48) | 643.63(442.85,927.29) | 1.05(0.78,1.33) |  | 59.33(25.44,123.56) | 5.56(2.39,11.56) | 1.06(0.79,1.33) |
| Guyana | 224.04(152.76,314.89) | 58.12(39.35,81.73) | 1.16(1.07,1.25) |  | 6222.82(4190.25,8892.01) | 1517.95(1022.21,2169.77) | 1.17(1.08,1.27) |  | 54.76(23.66,117.03) | 13.32(5.77,28.50) | 1.18(1.06,1.30) |
| Haiti | 2845.32(1961.19,4082.75) | 38.13(26.31,54.71) | 0.83(0.79,0.88) |  | 69762.78(47737.66,98320.58) | 976.99(669.63,1376.63) | 0.83(0.78,0.88) |  | 601.70(260.16,1266.09) | 8.42(3.65,17.67) | 0.78(0.72,0.84) |
| Honduras | 4965.11(3366.75,7119.83) | 81.11(54.85,116.84) | 1.27(1.14,1.40) |  | 119557.62(82136.91,173794.04) | 2086.66(1429.96,3031.72) | 1.27(1.16,1.39) |  | 1033.95(452.54,2194.72) | 17.99(7.91,38.23) | 1.25(1.12,1.37) |
| Hungary | 239.60(165.53,348.51) | 8.50(5.68,12.68) | 0.67(0.60,0.74) |  | 9278.07(6131.77,14078.20) | 215.28(141.05,329.60) | 0.67(0.60,0.74) |  | 80.23(33.53,170.67) | 1.87(0.78,3.93) | 0.67(0.60,0.75) |
| Iceland | 187.66(129.03,262.58) | 146.71(100.65,205.53) | 0.76(0.70,0.82) |  | 6018.40(4220.77,8549.60) | 3733.33(2607.91,5311.92) | 0.74(0.69,0.79) |  | 53.76(24.26,111.08) | 33.43(15.10,68.93) | 0.73(0.68,0.78) |
| India | 346723.54(248263.77,476794.28) | 46.93(33.28,64.95) | 2.02(1.88,2.16) |  | 9664220.13(6823030.48,13535722.54) | 1269.76(896.84,1778.02) | 2.34(2.19,2.49) |  | 84358.36(37037.10,176822.88) | 11.07(4.86,23.20) | 2.28(2.14,2.42) |
| Indonesia | 143576.89(102491.88,199866.65) | 111.30(78.67,155.47) | 2.40(2.30,2.49) |  | 4368497.54(3082597.04,6153283.56) | 2876.58(2028.48,4056.86) | 2.39(2.30,2.48) |  | 38469.04(17224.49,79270.48) | 25.38(11.36,52.45) | 2.34(2.26,2.41) |
| Iran (Islamic Republic of) | 29890.34(21006.57,42244.60) | 84.97(59.63,120.59) | 1.40(1.03,1.76) |  | 1038574.21(724084.69,1468509.87) | 2218.89(1544.61,3143.54) | 1.37(1.01,1.74) |  | 9440.52(4204.96,19825.60) | 20.33(9.07,42.81) | 1.39(1.04,1.76) |
| Iraq | 18837.37(12928.40,26888.57) | 77.47(53.29,110.53) | 0.60(0.48,0.72) |  | 433287.91(292930.53,614282.48) | 2031.04(1375.42,2881.58) | 0.61(0.49,0.73) |  | 3819.79(1685.89,7892.01) | 17.84(7.89,36.82) | 0.60(0.48,0.72) |
| Ireland | 2635.26(1847.00,3786.62) | 135.89(95.25,194.96) | 0.68(0.58,0.77) |  | 81450.66(57035.92,115151.42) | 3456.86(2412.02,4875.75) | 0.66(0.56,0.75) |  | 722.18(324.10,1478.03) | 30.91(13.88,63.01) | 0.65(0.55,0.74) |
| Israel | 5511.08(3786.10,7772.26) | 124.98(85.87,176.13) | 0.74(0.65,0.84) |  | 141616.67(98543.03,200911.28) | 3154.91(2193.73,4470.66) | 0.73(0.65,0.82) |  | 1259.56(562.48,2601.25) | 28.12(12.55,58.11) | 0.72(0.63,0.80) |
| Italy | 51695.96(36308.03,72441.10) | 326.18(227.34,458.58) | -0.65(-0.79,-0.50) |  | 1974972.62(1396990.25,2743152.57) | 8113.16(5757.74,11265.85) | -0.43(-0.56,-0.30) |  | 17308.67(7773.82,36906.11) | 71.69(32.16,152.77) | -0.38(-0.51,-0.26) |
| Jamaica | 753.67(513.58,1060.03) | 58.91(39.66,82.96) | 0.96(0.89,1.03) |  | 23878.27(16204.86,34305.58) | 1537.25(1041.90,2211.88) | 0.97(0.89,1.04) |  | 211.32(90.34,449.88) | 13.60(5.83,28.88) | 0.97(0.89,1.04) |
| Japan | 87447.99(61827.73,123721.40) | 266.51(189.72,375.55) | 0.25(0.20,0.31) |  | 3234636.08(2310710.42,4540640.15) | 6334.11(4579.73,8798.66) | 0.20(0.15,0.24) |  | 27969.82(12709.01,56664.46) | 55.14(25.10,111.98) | 0.19(0.14,0.24) |
| Jordan | 5754.51(3975.20,8166.85) | 79.57(54.97,112.66) | 0.88(0.83,0.94) |  | 130388.92(91058.39,184647.31) | 2079.34(1454.02,2938.43) | 0.87(0.81,0.93) |  | 1156.00(501.14,2448.40) | 18.36(7.94,38.84) | 0.80(0.71,0.89) |
| Kazakhstan | 1716.49(1172.81,2430.02) | 20.32(13.97,28.66) | 1.19(1.16,1.22) |  | 49882.88(33773.18,70716.75) | 519.72(349.98,738.04) | 1.22(1.19,1.25) |  | 435.49(187.57,922.12) | 4.56(1.97,9.67) | 1.20(1.16,1.23) |
| Kenya | 9930.28(7000.10,14105.33) | 28.93(20.71,40.66) | 0.52(0.42,0.62) |  | 197985.63(137954.87,284628.13) | 745.75(525.02,1066.22) | 0.53(0.42,0.64) |  | 1716.72(732.13,3614.92) | 6.42(2.75,13.58) | 0.54(0.43,0.65) |
| Kiribati | 55.99(39.14,79.19) | 78.42(55.09,110.62) | 1.11(0.89,1.33) |  | 1307.90(912.92,1839.80) | 2019.01(1411.32,2839.09) | 1.12(0.90,1.34) |  | 11.45(5.06,23.69) | 17.64(7.79,36.54) | 1.12(0.90,1.34) |
| Kuwait | 1591.09(1106.09,2267.06) | 103.15(71.59,147.51) | 0.80(0.74,0.86) |  | 78719.95(54159.35,111727.19) | 2710.67(1867.51,3844.20) | 0.80(0.74,0.86) |  | 688.07(302.47,1416.00) | 23.93(10.50,49.37) | 0.79(0.74,0.85) |
| Kyrgyzstan | 560.66(373.38,802.06) | 15.96(10.75,22.84) | 0.46(0.38,0.53) |  | 14244.12(9519.58,20750.52) | 406.84(272.05,592.79) | 0.45(0.37,0.53) |  | 124.42(51.77,269.78) | 3.55(1.48,7.69) | 0.46(0.38,0.53) |
| Lao People's Democratic Republic | 3822.97(2706.67,5367.52) | 95.13(67.72,133.06) | 2.22(2.16,2.28) |  | 97477.72(68812.95,140227.03) | 2434.47(1718.64,3501.88) | 2.23(2.17,2.29) |  | 860.46(384.62,1767.18) | 21.43(9.60,44.10) | 2.23(2.17,2.29) |
| Latvia | 61.90(43.77,87.72) | 11.05(7.72,15.80) | 1.03(0.96,1.10) |  | 2222.83(1532.54,3192.61) | 277.56(189.97,403.99) | 1.07(1.00,1.15) |  | 19.33(7.99,41.39) | 2.44(1.02,5.21) | 1.05(0.97,1.12) |
| Lebanon | 1996.52(1381.87,2842.67) | 88.40(61.19,125.89) | 0.88(0.86,0.90) |  | 69485.10(48252.29,99847.95) | 2333.51(1616.60,3359.94) | 0.89(0.87,0.91) |  | 603.37(260.88,1242.77) | 20.34(8.84,41.92) | 0.90(0.88,0.92) |
| Lesotho | 438.45(301.43,629.23) | 36.43(25.21,52.58) | 1.25(1.12,1.38) |  | 9573.54(6489.29,13849.82) | 934.81(634.27,1354.64) | 1.26(1.13,1.39) |  | 83.34(36.21,184.25) | 8.08(3.52,17.79) | 1.25(1.12,1.38) |
| Liberia | 1007.31(700.77,1458.13) | 27.66(19.31,39.77) | 1.34(1.22,1.46) |  | 19961.54(13688.22,29350.59) | 711.82(489.58,1042.57) | 1.40(1.28,1.52) |  | 170.03(73.54,351.26) | 6.03(2.60,12.44) | 1.36(1.25,1.47) |
| Libya | 2778.77(1920.58,3948.04) | 84.61(58.00,121.75) | 0.44(0.40,0.49) |  | 88291.27(61276.50,126378.55) | 2217.09(1533.97,3177.09) | 0.46(0.41,0.51) |  | 778.16(337.73,1588.44) | 19.61(8.52,39.99) | 0.46(0.41,0.51) |
| Lithuania | 85.51(61.57,122.37) | 10.68(7.48,15.60) | 1.24(1.16,1.31) |  | 3154.16(2197.45,4667.15) | 267.43(184.63,401.79) | 1.30(1.21,1.38) |  | 27.42(11.30,58.21) | 2.34(0.95,5.02) | 1.26(1.18,1.34) |
| Luxembourg | 270.78(186.40,383.14) | 142.81(98.04,203.20) | 0.81(0.75,0.87) |  | 11390.03(8020.95,16079.32) | 3617.44(2530.86,5086.89) | 0.80(0.74,0.85) |  | 101.37(44.76,210.34) | 32.39(14.42,67.35) | 0.79(0.73,0.84) |
| Madagascar | 4264.41(2937.15,6112.58) | 21.82(15.25,31.05) | 0.51(0.46,0.56) |  | 80942.70(55276.33,118989.19) | 555.02(380.82,810.24) | 0.53(0.48,0.58) |  | 718.34(308.70,1468.93) | 4.89(2.09,9.97) | 0.57(0.51,0.62) |
| Malawi | 4248.73(2984.65,6103.36) | 28.76(20.26,41.04) | 0.50(0.40,0.61) |  | 72724.25(49813.25,105705.54) | 720.04(497.66,1039.89) | 0.45(0.32,0.57) |  | 625.69(276.63,1277.04) | 6.15(2.71,12.74) | 0.43(0.31,0.54) |
| Malaysia | 23814.38(16714.09,33137.16) | 161.50(112.86,226.19) | 1.90(1.75,2.06) |  | 701853.94(489841.82,1003730.48) | 4125.99(2878.52,5885.36) | 1.89(1.74,2.05) |  | 6067.75(2694.87,12347.25) | 35.66(15.84,72.57) | 1.89(1.73,2.05) |
| Maldives | 226.96(158.22,323.27) | 128.78(89.70,183.56) | 3.47(3.19,3.76) |  | 7696.13(5304.16,11085.85) | 3297.11(2261.38,4751.03) | 3.41(3.13,3.70) |  | 67.88(29.92,147.11) | 29.20(12.85,63.36) | 3.41(3.13,3.70) |
| Mali | 3708.50(2532.36,5317.97) | 22.87(15.87,32.43) | 1.20(1.01,1.40) |  | 63796.99(43036.57,92385.73) | 579.36(394.12,836.53) | 1.26(1.04,1.47) |  | 554.94(241.82,1183.18) | 4.99(2.18,10.79) | 1.25(1.02,1.47) |
| Malta | 160.71(110.06,228.13) | 141.40(95.89,201.87) | 0.94(0.79,1.09) |  | 6848.86(4772.90,9748.92) | 3600.05(2508.73,5102.58) | 0.91(0.77,1.05) |  | 60.86(27.47,124.03) | 32.21(14.45,65.51) | 0.89(0.76,1.03) |
| Marshall Islands | 25.16(17.56,35.05) | 76.66(53.65,106.94) | 1.47(1.29,1.66) |  | 590.39(408.05,824.07) | 1974.44(1363.98,2758.18) | 1.48(1.30,1.66) |  | 5.16(2.24,10.81) | 17.24(7.50,36.11) | 1.47(1.29,1.65) |
| Mauritania | 973.77(674.40,1397.44) | 32.51(22.66,46.17) | 0.72(0.54,0.91) |  | 18290.52(12523.84,26298.87) | 840.80(576.26,1205.45) | 0.76(0.57,0.96) |  | 161.01(68.97,326.59) | 7.34(3.15,14.94) | 0.76(0.57,0.96) |
| Mauritius | 699.28(496.48,976.86) | 147.10(103.43,208.27) | 1.68(1.56,1.80) |  | 24029.62(16784.23,34201.96) | 3769.91(2642.56,5384.13) | 1.68(1.56,1.80) |  | 209.50(91.94,440.69) | 32.96(14.50,69.47) | 1.67(1.55,1.79) |
| Mexico | 89661.69(63147.57,124689.16) | 141.45(99.10,197.25) | -0.53(-0.76,-0.29) |  | 2553118.52(1801191.16,3534174.73) | 3622.47(2553.10,5009.74) | -0.51(-0.75,-0.28) |  | 22018.12(9764.82,46551.95) | 31.26(13.85,66.05) | -0.53(-0.77,-0.29) |
| Micronesia (Federated States of) | 51.21(35.87,72.04) | 84.43(59.11,119.36) | 0.96(0.75,1.16) |  | 1147.44(803.47,1639.35) | 2173.82(1520.35,3095.74) | 0.97(0.76,1.17) |  | 10.08(4.50,20.74) | 19.04(8.50,39.28) | 0.96(0.75,1.17) |
| Monaco | 13.91(9.51,19.73) | 143.38(98.27,204.74) | 0.50(0.43,0.57) |  | 529.34(366.26,747.10) | 3641.66(2511.44,5143.38) | 0.48(0.41,0.55) |  | 4.69(2.12,9.87) | 32.58(14.67,68.30) | 0.46(0.40,0.53) |
| Mongolia | 276.10(188.77,395.99) | 18.08(12.37,25.90) | 1.21(1.16,1.26) |  | 7888.07(5312.87,11227.35) | 460.34(310.36,656.75) | 1.25(1.20,1.30) |  | 68.88(28.62,150.47) | 4.04(1.69,8.81) | 1.26(1.21,1.31) |
| Montenegro | 18.75(12.82,27.26) | 8.54(5.77,12.60) | 1.00(0.92,1.08) |  | 629.41(414.07,930.00) | 216.34(141.41,323.08) | 1.02(0.94,1.11) |  | 5.47(2.20,11.59) | 1.89(0.76,4.03) | 1.03(0.95,1.12) |
| Morocco | 13920.95(9757.56,20090.55) | 77.28(54.07,111.57) | 0.91(0.89,0.93) |  | 396061.13(272630.40,566961.03) | 2030.18(1397.84,2905.20) | 1.00(0.96,1.05) |  | 3516.54(1567.10,7249.74) | 18.05(8.04,37.17) | 0.96(0.92,1.00) |
| Mozambique | 6138.26(4201.24,8777.84) | 27.65(19.07,39.32) | 1.29(1.21,1.37) |  | 109182.43(73741.88,159482.82) | 711.86(483.64,1033.43) | 1.34(1.25,1.43) |  | 939.55(395.41,1916.63) | 6.07(2.57,12.45) | 1.33(1.24,1.42) |
| Myanmar | 29204.41(20728.61,41144.10) | 97.89(69.86,137.51) | 2.79(2.65,2.93) |  | 758041.37(531570.86,1064624.72) | 2487.69(1744.39,3493.60) | 2.77(2.63,2.91) |  | 6698.33(2971.95,13839.46) | 21.97(9.74,45.39) | 2.78(2.64,2.92) |
| Namibia | 522.14(365.12,748.65) | 34.88(24.46,49.90) | 0.91(0.78,1.04) |  | 11832.79(8097.12,16973.73) | 885.34(609.78,1270.79) | 0.92(0.79,1.05) |  | 103.20(45.69,210.71) | 7.69(3.41,15.66) | 0.88(0.74,1.02) |
| Nauru | 6.57(4.59,9.39) | 98.26(68.62,140.24) | 1.06(0.99,1.13) |  | 145.68(101.58,211.27) | 2531.50(1764.66,3680.18) | 1.06(0.99,1.13) |  | 1.28(0.56,2.64) | 22.10(9.74,45.92) | 1.06(0.99,1.13) |
| Nepal | 4855.03(3368.11,6762.04) | 26.39(18.33,36.92) | 1.85(1.79,1.91) |  | 119833.17(82473.80,171688.60) | 655.33(450.84,937.30) | 1.82(1.77,1.87) |  | 1076.03(455.11,2227.43) | 5.86(2.48,12.16) | 1.82(1.77,1.86) |
| Netherlands | 6746.48(4690.74,9664.33) | 123.50(85.50,177.57) | 0.71(0.66,0.76) |  | 234128.23(162311.63,332033.58) | 3147.42(2172.38,4452.61) | 0.70(0.65,0.74) |  | 2092.03(917.22,4388.81) | 28.23(12.41,58.90) | 0.68(0.63,0.73) |
| New Zealand | 4381.92(3113.90,6041.78) | 236.50(167.69,326.85) | -0.21(-0.34,-0.08) |  | 137891.01(99422.28,187997.77) | 5689.13(4094.50,7762.63) | -0.11(-0.23,0.00) |  | 1199.22(550.69,2502.83) | 49.57(22.70,103.17) | -0.10(-0.21,0.01) |
| Nicaragua | 3142.27(2105.89,4543.95) | 87.88(58.85,127.37) | 1.04(0.95,1.14) |  | 82216.23(54856.32,117750.48) | 2246.36(1501.49,3214.56) | 1.05(0.96,1.14) |  | 715.82(312.52,1548.77) | 19.53(8.54,42.28) | 1.04(0.94,1.13) |
| Niger | 3547.11(2473.24,5103.77) | 20.38(14.43,28.80) | 0.87(0.73,1.01) |  | 55429.56(38278.32,80303.97) | 517.15(357.86,748.60) | 0.91(0.76,1.06) |  | 484.08(199.05,1012.41) | 4.47(1.88,9.34) | 0.83(0.69,0.98) |
| Nigeria | 46649.81(32689.38,66406.83) | 28.39(20.27,39.84) | 0.64(0.48,0.81) |  | 848802.62(590703.73,1212326.10) | 739.31(518.44,1052.63) | 0.74(0.56,0.91) |  | 7410.20(3180.20,15722.56) | 6.39(2.75,13.68) | 0.76(0.58,0.94) |
| Niue | 0.82(0.58,1.18) | 112.47(78.48,161.45) | 1.27(1.11,1.43) |  | 22.42(15.69,32.09) | 2895.05(2019.02,4151.71) | 1.27(1.11,1.43) |  | 0.20(0.09,0.41) | 25.32(11.30,52.79) | 1.27(1.10,1.43) |
| North Macedonia | 53.79(36.49,77.55) | 7.77(5.16,11.52) | 1.13(1.06,1.20) |  | 2109.04(1387.72,3169.96) | 196.09(127.73,299.07) | 1.15(1.08,1.22) |  | 18.23(7.81,38.58) | 1.71(0.73,3.62) | 1.15(1.07,1.22) |
| Northern Mariana Islands | 23.96(16.55,33.96) | 106.78(74.03,150.86) | 0.67(0.51,0.83) |  | 626.96(431.87,881.12) | 2752.40(1891.74,3874.16) | 0.66(0.50,0.82) |  | 5.47(2.40,11.56) | 24.12(10.51,51.12) | 0.66(0.50,0.82) |
| Norway | 2287.24(1616.52,3227.92) | 125.20(88.34,177.42) | 0.32(0.21,0.42) |  | 78878.53(55520.87,112443.90) | 3217.26(2259.05,4591.03) | 0.32(0.22,0.43) |  | 702.64(312.90,1448.01) | 28.80(12.89,59.17) | 0.32(0.22,0.42) |
| Oman | 1642.26(1122.40,2355.76) | 91.72(63.06,129.69) | 1.73(1.65,1.82) |  | 49269.59(33720.92,70294.30) | 2410.09(1651.57,3437.57) | 1.75(1.66,1.83) |  | 432.43(187.83,898.89) | 21.20(9.25,44.09) | 1.75(1.66,1.84) |
| Pakistan | 42077.19(29621.89,59560.90) | 30.39(21.44,42.85) | 0.69(0.58,0.80) |  | 933701.62(636727.64,1334539.13) | 765.75(526.76,1091.90) | 0.55(0.40,0.69) |  | 8439.21(3618.35,18337.42) | 6.89(2.97,14.96) | 0.56(0.39,0.73) |
| Palau | 7.53(5.15,10.84) | 112.01(76.55,161.10) | 1.04(0.88,1.21) |  | 218.20(150.72,312.02) | 2887.70(1990.57,4126.06) | 1.04(0.87,1.20) |  | 1.89(0.83,3.86) | 25.24(11.10,51.37) | 1.04(0.87,1.20) |
| Palestine | 2401.70(1650.27,3363.94) | 73.97(50.93,103.68) | 0.74(0.69,0.79) |  | 51397.09(35008.67,72817.76) | 1942.17(1327.01,2740.61) | 0.75(0.70,0.80) |  | 455.57(205.91,966.34) | 17.10(7.73,36.17) | 0.75(0.70,0.80) |
| Panama | 1867.64(1270.28,2728.17) | 90.09(61.25,131.72) | 1.36(1.27,1.46) |  | 50374.87(34715.37,73503.88) | 2331.37(1607.36,3400.98) | 1.36(1.27,1.46) |  | 434.86(188.54,946.93) | 20.12(8.73,43.82) | 1.35(1.26,1.45) |
| Papua New Guinea | 3693.08(2565.94,5174.03) | 64.43(44.85,90.65) | 1.00(0.83,1.17) |  | 86753.67(60039.61,124936.87) | 1637.42(1134.30,2363.63) | 1.00(0.83,1.18) |  | 752.70(326.34,1588.10) | 14.19(6.16,29.90) | 1.01(0.83,1.19) |
| Paraguay | 883.03(607.77,1278.29) | 23.19(15.89,33.63) | 1.62(1.56,1.68) |  | 23063.60(15598.86,33566.16) | 604.40(409.30,878.16) | 1.67(1.61,1.73) |  | 202.80(85.97,422.95) | 5.30(2.25,11.07) | 1.65(1.59,1.71) |
| Peru | 21143.22(14330.33,30538.89) | 129.39(87.30,188.00) | 0.96(0.91,1.01) |  | 627726.45(429269.94,908335.01) | 3241.41(2215.89,4687.01) | 1.22(1.18,1.26) |  | 5405.22(2339.78,11490.03) | 27.92(12.06,59.40) | 1.21(1.17,1.24) |
| Philippines | 67789.02(48104.46,94089.37) | 108.84(77.28,151.59) | 1.97(1.87,2.06) |  | 1658829.46(1159118.31,2353345.26) | 2801.89(1957.64,3975.80) | 2.17(2.03,2.31) |  | 14741.10(6394.37,30412.00) | 24.85(10.81,51.30) | 2.15(2.02,2.28) |
| Poland | 1206.07(881.81,1614.69) | 10.53(7.59,14.17) | -0.08(-0.16,0.00) |  | 47767.16(33886.08,64540.92) | 269.48(190.73,368.48) | -0.01(-0.08,0.05) |  | 414.80(184.26,852.29) | 2.36(1.05,4.77) | -0.02(-0.08,0.04) |
| Portugal | 3817.52(2607.44,5390.12) | 128.22(87.30,180.67) | 0.76(0.57,0.95) |  | 153295.73(105684.98,217610.86) | 3268.08(2250.48,4623.77) | 0.74(0.56,0.92) |  | 1358.16(627.26,2727.64) | 29.25(13.51,58.84) | 0.74(0.55,0.92) |
| Puerto Rico | 972.60(656.77,1399.20) | 87.35(59.06,125.31) | 0.82(0.74,0.90) |  | 34174.37(22806.88,49848.03) | 2271.06(1510.57,3321.18) | 0.82(0.74,0.90) |  | 300.39(133.02,623.41) | 20.05(8.88,41.54) | 0.82(0.74,0.90) |
| Qatar | 720.78(491.23,1046.59) | 101.08(68.65,146.01) | 0.54(0.50,0.58) |  | 29513.70(20102.30,42804.06) | 2660.35(1810.21,3885.75) | 0.54(0.50,0.58) |  | 258.18(111.66,529.74) | 23.43(10.24,48.28) | 0.53(0.49,0.57) |
| Republic of Korea | 17390.88(12118.39,24770.88) | 130.61(91.77,185.36) | 1.14(0.79,1.49) |  | 749041.19(521395.30,1062722.41) | 3117.94(2187.29,4453.20) | 1.11(0.77,1.45) |  | 6500.21(2979.29,13074.02) | 27.23(12.49,55.05) | 1.10(0.76,1.44) |
| Republic of Moldova | 110.64(78.49,153.81) | 9.90(6.83,14.06) | 1.44(1.30,1.58) |  | 4464.96(3085.66,6491.15) | 247.11(167.49,364.08) | 1.51(1.36,1.66) |  | 39.24(16.29,84.65) | 2.20(0.91,4.71) | 1.49(1.35,1.62) |
| Romania | 500.80(340.11,738.12) | 8.21(5.55,12.24) | 1.21(1.16,1.26) |  | 17218.93(11343.60,26103.50) | 207.94(136.20,318.30) | 1.23(1.18,1.28) |  | 148.95(61.67,309.85) | 1.81(0.75,3.80) | 1.24(1.19,1.29) |
| Russian Federation | 5581.88(3998.22,7753.70) | 11.18(7.86,15.88) | 0.94(0.90,0.98) |  | 192213.74(134056.69,274334.90) | 273.16(189.03,391.57) | 0.99(0.95,1.04) |  | 1676.85(693.03,3513.19) | 2.41(0.99,5.02) | 0.99(0.95,1.04) |
| Rwanda | 2483.29(1708.63,3612.23) | 28.51(19.76,41.16) | 0.86(0.80,0.91) |  | 51083.48(34967.20,73761.69) | 719.98(494.75,1039.74) | 0.82(0.76,0.88) |  | 443.66(189.82,950.02) | 6.23(2.68,13.28) | 0.79(0.72,0.87) |
| Saint Kitts and Nevis | 15.49(10.77,22.31) | 70.38(47.73,102.70) | 0.90(0.82,0.98) |  | 572.36(390.26,832.55) | 1831.33(1243.87,2655.58) | 0.90(0.81,0.98) |  | 5.03(2.24,10.44) | 16.17(7.20,33.53) | 0.90(0.82,0.99) |
| Saint Lucia | 37.65(26.03,52.91) | 57.81(39.37,82.03) | 0.68(0.54,0.82) |  | 1377.28(942.25,1928.74) | 1501.80(1022.68,2110.44) | 0.68(0.53,0.82) |  | 12.11(5.34,25.36) | 13.27(5.86,27.62) | 0.67(0.53,0.82) |
| Saint Vincent and the Grenadines | 29.76(20.02,42.07) | 59.34(39.91,84.10) | 1.36(1.27,1.46) |  | 862.92(583.26,1232.99) | 1539.91(1040.44,2200.11) | 1.38(1.28,1.48) |  | 7.61(3.38,16.37) | 13.61(6.01,29.23) | 1.37(1.27,1.47) |
| Samoa | 118.71(81.81,171.55) | 94.35(65.04,135.51) | 0.86(0.73,0.99) |  | 2401.49(1649.68,3416.92) | 2430.46(1677.51,3464.56) | 0.86(0.73,0.99) |  | 21.07(9.48,44.08) | 21.25(9.51,44.65) | 0.86(0.73,0.99) |
| San Marino | 13.66(9.44,19.01) | 138.58(94.84,193.99) | 0.54(0.45,0.62) |  | 506.89(347.34,709.99) | 3519.75(2397.78,4919.30) | 0.52(0.43,0.60) |  | 4.49(2.00,9.46) | 31.49(13.97,65.80) | 0.50(0.42,0.59) |
| Sao Tome and Principe | 42.90(30.07,61.86) | 29.77(20.97,42.74) | 1.02(0.81,1.22) |  | 856.24(588.78,1231.70) | 756.38(522.43,1083.55) | 1.05(0.85,1.26) |  | 7.42(3.25,15.31) | 6.53(2.85,13.49) | 1.06(0.85,1.27) |
| Saudi Arabia | 13424.19(9203.31,18972.80) | 99.30(67.62,141.76) | 1.22(1.19,1.24) |  | 527576.33(362950.60,757709.60) | 2608.01(1796.32,3769.73) | 1.22(1.20,1.24) |  | 4625.85(2084.79,9737.69) | 22.95(10.33,48.13) | 1.22(1.20,1.24) |
| Senegal | 2746.02(1881.63,3950.09) | 27.06(18.73,38.68) | 0.48(0.36,0.61) |  | 54552.69(37552.20,78335.20) | 691.99(476.55,995.18) | 0.51(0.39,0.64) |  | 477.97(207.48,1018.12) | 6.02(2.62,12.94) | 0.51(0.39,0.64) |
| Serbia | 237.90(162.21,338.81) | 7.84(5.25,11.29) | 1.09(1.02,1.15) |  | 8136.77(5374.23,11804.13) | 198.24(130.49,289.42) | 1.10(1.04,1.17) |  | 70.88(29.15,151.08) | 1.73(0.72,3.68) | 1.10(1.04,1.17) |
| Seychelles | 58.27(40.89,81.71) | 137.81(96.61,193.50) | 1.10(1.05,1.16) |  | 1728.21(1206.91,2450.28) | 3523.90(2459.90,5007.50) | 1.08(1.03,1.14) |  | 15.13(6.71,31.30) | 30.96(13.71,64.18) | 1.08(1.02,1.13) |
| Sierra Leone | 1624.61(1116.47,2356.54) | 27.70(19.15,40.03) | 1.47(1.33,1.60) |  | 32666.83(22104.79,48130.79) | 713.61(484.16,1047.16) | 1.54(1.39,1.68) |  | 280.56(119.94,600.08) | 6.08(2.60,12.95) | 1.52(1.37,1.67) |
| Singapore | 2033.24(1407.67,2951.38) | 154.59(106.48,225.31) | 1.58(1.42,1.73) |  | 111651.03(78448.44,161172.91) | 3694.70(2599.40,5295.41) | 1.54(1.39,1.69) |  | 966.38(454.57,2037.03) | 32.24(14.90,68.63) | 1.53(1.38,1.68) |
| Slovakia | 136.64(96.01,198.33) | 8.29(5.71,12.21) | 1.06(1.02,1.10) |  | 5355.00(3557.94,8021.01) | 209.94(138.84,314.83) | 1.07(1.02,1.11) |  | 46.42(18.32,105.23) | 1.83(0.73,4.19) | 1.07(1.03,1.11) |
| Slovenia | 51.84(34.80,74.97) | 8.89(5.95,12.98) | 1.11(1.01,1.21) |  | 1916.83(1243.44,2850.68) | 223.83(145.40,331.38) | 1.16(1.05,1.27) |  | 16.37(6.93,34.63) | 1.92(0.82,4.06) | 1.13(1.03,1.24) |
| Solomon Islands | 293.55(200.70,414.36) | 71.08(48.69,100.38) | 1.22(0.98,1.47) |  | 6327.66(4382.91,8803.60) | 1827.09(1265.61,2539.07) | 1.23(0.98,1.48) |  | 55.51(24.64,118.62) | 15.99(7.10,34.09) | 1.23(0.99,1.48) |
| Somalia | 3077.54(2136.00,4496.51) | 21.92(15.42,31.51) | 0.57(0.52,0.62) |  | 54013.01(37137.14,80152.32) | 556.85(381.31,821.91) | 0.59(0.55,0.64) |  | 470.86(197.98,999.33) | 4.82(2.02,10.22) | 0.62(0.57,0.67) |
| South Africa | 13170.77(9223.67,18578.58) | 46.64(32.60,66.11) | 0.94(0.88,1.00) |  | 369432.20(252296.50,524096.96) | 1179.02(803.60,1670.77) | 0.93(0.86,1.01) |  | 3198.11(1370.06,6653.58) | 10.22(4.40,21.26) | 0.89(0.82,0.97) |
| South Sudan | 1564.30(1066.92,2272.68) | 23.01(16.04,32.97) | 0.17(0.07,0.27) |  | 27377.44(18722.11,39864.12) | 586.39(401.64,846.72) | 0.18(0.07,0.29) |  | 238.89(103.20,512.17) | 5.09(2.19,10.82) | 0.20(0.09,0.30) |
| Spain | 17911.06(12395.82,25474.14) | 132.93(91.49,189.51) | 0.60(0.47,0.73) |  | 661982.69(463317.26,921945.29) | 3307.02(2315.93,4612.92) | 0.66(0.53,0.79) |  | 5803.44(2605.98,12243.40) | 29.30(13.32,61.49) | 0.63(0.49,0.76) |
| Sri Lanka | 12914.94(9127.41,17884.30) | 124.05(87.31,172.30) | 1.88(1.66,2.10) |  | 360441.94(254149.60,503919.08) | 3164.18(2231.82,4418.47) | 1.87(1.65,2.09) |  | 3158.74(1399.84,6284.37) | 27.80(12.32,55.29) | 1.85(1.63,2.06) |
| Sudan | 19399.16(13434.16,27299.16) | 68.77(47.85,96.50) | 1.92(1.77,2.08) |  | 415561.94(287134.84,592393.25) | 1817.65(1262.35,2594.65) | 1.96(1.81,2.12) |  | 3598.14(1621.65,7495.69) | 15.67(7.05,32.71) | 1.94(1.77,2.10) |
| Suriname | 165.84(112.60,234.31) | 61.38(41.59,86.89) | 0.99(0.96,1.03) |  | 4686.80(3190.59,6702.62) | 1595.49(1084.73,2279.93) | 0.99(0.96,1.03) |  | 41.17(18.11,87.86) | 14.04(6.19,30.03) | 0.99(0.95,1.03) |
| Sweden | 3463.70(2438.03,4906.52) | 101.12(70.76,143.70) | 0.52(0.27,0.78) |  | 116224.62(81057.16,164271.65) | 2606.76(1811.07,3683.24) | 0.49(0.23,0.75) |  | 1032.61(465.91,2121.43) | 23.32(10.52,47.78) | 0.48(0.23,0.73) |
| Switzerland | 3008.92(2108.45,4291.55) | 122.96(85.77,176.87) | 0.41(0.40,0.43) |  | 124099.20(86042.96,175921.00) | 3125.63(2182.43,4408.64) | 0.41(0.39,0.43) |  | 1102.00(492.00,2261.99) | 27.95(12.46,57.00) | 0.40(0.39,0.42) |
| Syrian Arab Republic | 7310.15(5119.47,10484.15) | 77.22(53.73,110.46) | 0.96(0.89,1.04) |  | 158407.94(109681.36,226455.55) | 2026.43(1402.83,2878.71) | 0.99(0.91,1.07) |  | 1383.18(613.74,2874.08) | 17.70(7.85,36.90) | 0.98(0.90,1.07) |
| Taiwan (Province of China) | 5631.12(4047.85,7549.93) | 96.73(69.47,131.02) | 1.80(1.71,1.89) |  | 281539.00(199611.41,384540.58) | 2489.94(1762.39,3400.01) | 1.79(1.70,1.87) |  | 2416.42(1055.78,4838.76) | 21.51(9.46,43.18) | 1.79(1.70,1.87) |
| Tajikistan | 743.82(504.25,1075.79) | 13.91(9.48,19.94) | 0.88(0.82,0.95) |  | 17975.90(11937.70,26236.95) | 348.14(231.14,507.29) | 0.90(0.83,0.96) |  | 160.49(65.30,346.47) | 3.10(1.26,6.67) | 0.93(0.86,0.99) |
| Thailand | 29434.96(20972.88,41107.52) | 136.04(96.20,190.05) | 2.26(2.11,2.42) |  | 1145788.14(805847.95,1629052.61) | 3482.20(2427.75,4950.67) | 2.26(2.10,2.41) |  | 10121.70(4512.26,21216.90) | 30.98(13.79,65.46) | 2.22(2.07,2.37) |
| Timor-Leste | 744.44(529.54,1074.75) | 80.27(57.49,116.88) | 1.98(1.86,2.09) |  | 14238.87(10026.86,20540.65) | 2052.11(1441.38,2978.82) | 1.97(1.86,2.08) |  | 127.30(55.32,257.76) | 18.19(7.98,36.77) | 1.98(1.87,2.09) |
| Togo | 1353.64(940.51,1965.70) | 26.21(18.27,37.77) | 1.05(0.89,1.21) |  | 29182.17(19921.99,42929.40) | 669.85(459.08,982.57) | 1.09(0.92,1.27) |  | 254.07(109.00,540.76) | 5.81(2.50,12.39) | 1.10(0.93,1.27) |
| Tokelau | 0.74(0.51,1.06) | 101.55(70.21,144.88) | 1.49(1.34,1.64) |  | 17.05(11.76,24.45) | 2616.30(1803.44,3743.89) | 1.50(1.35,1.65) |  | 0.15(0.07,0.31) | 22.85(10.43,47.33) | 1.49(1.34,1.64) |
| Tonga | 66.71(45.44,96.36) | 108.25(73.80,156.06) | 0.69(0.48,0.91) |  | 1428.88(977.50,2032.00) | 2790.79(1907.11,3970.77) | 0.69(0.48,0.91) |  | 12.51(5.48,25.77) | 24.38(10.65,50.19) | 0.69(0.47,0.90) |
| Trinidad and Tobago | 352.26(235.89,505.29) | 65.49(43.65,94.12) | 1.30(1.16,1.45) |  | 11664.29(7830.91,16720.29) | 1708.60(1152.71,2458.00) | 1.32(1.17,1.47) |  | 102.19(44.74,211.96) | 15.08(6.58,31.19) | 1.30(1.15,1.44) |
| Tunisia | 3844.36(2668.33,5457.31) | 79.26(54.87,112.67) | 1.22(1.17,1.26) |  | 128415.11(88919.52,181871.56) | 2082.09(1439.97,2956.93) | 1.22(1.18,1.26) |  | 1133.07(504.13,2370.67) | 18.48(8.25,38.75) | 1.19(1.15,1.24) |
| Turkey | 26768.25(18464.82,37876.43) | 74.78(51.54,106.27) | 1.23(1.16,1.30) |  | 849004.60(583999.91,1212199.10) | 1954.56(1341.83,2795.21) | 1.19(1.13,1.25) |  | 7466.54(3325.56,15703.12) | 17.25(7.69,36.33) | 1.08(1.00,1.15) |
| Turkmenistan | 493.01(339.03,706.79) | 19.54(13.43,28.02) | 1.22(1.19,1.25) |  | 12694.55(8533.95,18669.73) | 499.41(335.70,734.32) | 1.26(1.24,1.29) |  | 110.77(46.80,235.31) | 4.36(1.84,9.24) | 1.26(1.23,1.28) |
| Tuvalu | 5.91(4.10,8.36) | 91.62(63.54,130.16) | 1.26(1.08,1.44) |  | 138.60(95.34,198.07) | 2359.26(1626.10,3363.82) | 1.27(1.09,1.45) |  | 1.21(0.55,2.57) | 20.62(9.44,43.69) | 1.29(1.11,1.46) |
| Uganda | 8104.55(5657.14,11650.40) | 26.55(18.65,37.74) | 0.73(0.70,0.75) |  | 141482.63(98389.42,205019.56) | 672.52(462.55,967.04) | 0.73(0.69,0.76) |  | 1224.76(525.73,2520.42) | 5.78(2.48,11.92) | 0.68(0.63,0.73) |
| Ukraine | 1396.65(1019.61,1945.88) | 9.84(6.90,14.14) | 0.73(0.66,0.79) |  | 50300.10(34527.26,72962.87) | 237.18(161.22,349.27) | 0.78(0.71,0.85) |  | 444.55(188.22,945.11) | 2.13(0.89,4.54) | 0.76(0.69,0.83) |
| United Arab Emirates | 2034.26(1397.03,2922.47) | 92.60(63.36,132.81) | 0.85(0.73,0.96) |  | 83029.72(57477.25,119902.72) | 2433.55(1675.23,3522.80) | 0.85(0.73,0.97) |  | 716.67(312.61,1500.57) | 21.37(9.46,44.43) | 0.84(0.73,0.96) |
| United Kingdom | 34188.22(24138.90,47823.28) | 150.78(106.22,211.32) | 0.49(0.35,0.63) |  | 1193322.65(836035.90,1698024.04) | 3835.91(2672.27,5436.12) | 0.48(0.35,0.61) |  | 10601.61(4786.99,21948.98) | 34.25(15.52,70.62) | 0.47(0.34,0.60) |
| United Republic of Tanzania | 11879.41(8174.02,16805.79) | 29.83(20.77,42.01) | 0.63(0.54,0.72) |  | 229887.01(157473.70,328170.66) | 766.13(524.33,1092.09) | 0.63(0.54,0.72) |  | 1970.17(876.48,4073.28) | 6.54(2.92,13.54) | 0.62(0.54,0.71) |
| United States of America | 194555.53(142842.67,258038.33) | 157.14(116.75,206.42) | -0.65(-1.19,-0.12) |  | 6051074.95(4526937.14,7901919.81) | 3935.93(2940.53,5138.60) | -0.60(-1.14,-0.06) |  | 53388.06(24521.81,107618.81) | 34.83(16.07,70.21) | -0.62(-1.15,-0.08) |
| United States Virgin Islands | 23.48(15.84,33.78) | 83.82(56.13,120.67) | 0.71(0.61,0.82) |  | 758.39(504.94,1082.77) | 2177.74(1449.14,3121.48) | 0.71(0.61,0.82) |  | 6.65(2.91,14.16) | 19.22(8.40,41.13) | 0.70(0.60,0.81) |
| Uruguay | 1051.25(738.06,1515.15) | 77.63(54.72,112.02) | 1.61(1.42,1.81) |  | 33092.87(23318.20,48297.80) | 1968.43(1386.43,2858.94) | 1.61(1.42,1.81) |  | 291.33(126.74,581.69) | 17.38(7.59,34.85) | 1.60(1.40,1.80) |
| Uzbekistan | 2981.71(2069.36,4124.50) | 19.28(13.34,26.71) | 1.13(1.01,1.26) |  | 88482.21(59929.38,124366.42) | 491.56(333.67,691.99) | 1.15(1.03,1.28) |  | 770.77(329.65,1677.79) | 4.29(1.84,9.33) | 1.12(0.99,1.25) |
| Vanuatu | 142.37(96.91,206.02) | 77.93(53.18,112.57) | 1.21(1.16,1.27) |  | 3185.83(2156.22,4658.93) | 2007.65(1362.31,2935.24) | 1.22(1.16,1.27) |  | 27.99(12.44,59.89) | 17.58(7.79,37.45) | 1.21(1.16,1.27) |
| Venezuela (Bolivarian Republic of) | 12226.37(8482.93,17618.23) | 98.33(68.01,141.95) | 0.67(0.61,0.74) |  | 348798.09(235439.80,507764.27) | 2531.18(1709.99,3685.17) | 0.67(0.60,0.74) |  | 3027.90(1318.05,6350.49) | 22.06(9.61,46.27) | 0.66(0.59,0.73) |
| Viet Nam | 37473.61(26110.67,52256.84) | 90.74(63.35,127.03) | 2.80(2.62,2.98) |  | 1183045.17(824669.27,1688238.31) | 2281.42(1589.77,3247.08) | 2.81(2.63,2.99) |  | 10342.46(4400.17,21620.35) | 20.02(8.54,41.66) | 2.77(2.60,2.95) |
| Yemen | 11282.53(7615.29,16305.19) | 49.82(33.80,71.41) | 0.99(0.85,1.12) |  | 221143.19(147973.12,322243.65) | 1304.84(876.74,1890.88) | 0.99(0.86,1.12) |  | 1950.98(844.51,4188.36) | 11.45(4.94,24.53) | 0.99(0.86,1.13) |
| Zambia | 4669.43(3157.78,6691.20) | 34.33(23.39,48.84) | 0.54(0.49,0.58) |  | 88097.95(58546.67,130205.63) | 884.16(592.83,1290.42) | 0.51(0.46,0.57) |  | 762.38(326.72,1594.66) | 7.60(3.27,15.80) | 0.47(0.39,0.55) |
| Zimbabwe | 3386.46(2358.07,4811.41) | 32.29(22.62,45.66) | -0.07(-0.25,0.11) |  | 67564.15(46296.76,96606.88) | 823.01(564.10,1177.99) | -0.12(-0.31,0.06) |  | 584.02(249.22,1237.02) | 7.08(3.01,15.03) | -0.15(-0.34,0.05) |
