# Supplementary material for: Global, regional and national burden of polycystic ovary syndrome: historical trends from 1990 to 2021 and projections to 2035
Source: Front Endocrinol (Lausanne). 2026 Apr 1;17:1662823. doi: 10.3389/fendo.2026.1662823 (PMC13079045; doi:10.3389/fendo.2026.1662823)
Supplement: Supplementary file 8 [file DataSheet3.doc]

**Table S3.APC of polycystic ovary syndrome from 1990 to 2021 for the World and SDI.**

| Characteristics | | Incidence(95% CI) | |  | Prevalence(95% CI) | |  | DALYs(95% CI) | |
| --- | --- | --- | --- | --- | --- | --- | --- | --- | --- |
|  |  | APCs | P |  | APCs | P |  | APCs | P |
| Global |  | | | | | | | | |
|  | slope1 | 0.79(0.77,0.81) | * |  | 0.92(0.90,0.95) | * |  | 0.92(0.89,0.94) | * |
|  | slope2 | 0.48(0.38,0.58) | * |  | 0.30(0.22,0.37) | * |  | 0.28(0.22,0.35) | * |
|  | slope3 | 0.83(0.76,0.89) | * |  | 0.94(0.85,1.03) | * |  | 0.88(0.80,0.96) | * |
|  | slope4 | 1.10(0.99,1.20) | * |  | 1.15(1.06,1.24) | * |  | 1.13(1.04,1.21) | * |
| High SDI |  | | | | | | | | |
|  | slope1 | 0.53(0.44,0.62) | * |  | 0.62(0.53,0.70) | * |  | 0.75(0.52,0.99) | * |
|  | slope2 | -1.59(-1.77,-1.40) | * |  | -1.77(-1.99,-1.54) | * |  | -0.18(-0.84,0.49) |  |
|  | slope3 | 1.06(0.76,1.36) | * |  | 0.89(0.60,1.19) | * |  | -1.65(-2.04,-1.26) | * |
|  | slope4 | 3.40(2.99,3.81) | * |  | 3.09(2.69,3.48) | * |  | 2.00(1.77,2.24) | * |
| High middle SDI |  | | | | | | | | |
|  | slope1 | 0.34(0.26,0.43) | * |  | 0.92(0.77,1.07) | * |  | 1.00(0.89,1.11) | * |
|  | slope2 | 2.10(2.07,2.13) | * |  | 1.76(1.68,1.83) | * |  | 1.75(1.67,1.84) | * |
|  | slope3 | 0.95(0.78,1.12) | * |  | 0.89(0.80,0.98) | * |  | 0.87(0.78,0.95) | * |
|  | slope4 | -0.01(-0.25,0.23) |  |  | 1.18(1.14,1.22) | * |  | 1.17(1.13,1.20) | * |
| Middle SDI |  | | | | | | | | |
|  | slope1 | 2.10(1.90,2.30) | * |  | 1.87(1.78,1.97) | * |  | 2.05(2.01,2.08) | * |
|  | slope2 | 1.43(1.37,1.50) | * |  | 2.31(2.13,2.49) | * |  | 1.64(1.61,1.66) | * |
|  | slope3 | 1.81(1.77,1.85) | * |  | 1.66(1.64,1.68) | * |  | 1.39(1.26,1.51) | * |
|  | slope4 | 1.00(0.93,1.06) | * |  | 1.18(1.09,1.28) | * |  | 0.46(-0.08,1.01) |  |
| Low middle SDI |  | | | | | | | | |
|  | slope1 | 1.22(1.13,1.31) | * |  | 1.42(1.39,1.44) | * |  | 1.41(1.36,1.46) | * |
|  | slope2 | 1.33(1.22,1.44) | * |  | 2.21(2.14,2.29) | * |  | 1.64(1.37,1.91) | * |
|  | slope3 | 2.07(1.96,2.18) | * |  | 1.39(1.33,1.45) | * |  | 2.26(2.06,2.45) | * |
|  | slope4 | 1.25(1.19,1.31) | * |  | 0.23(-0.32,0.79) |  |  | 1.27(1.21,1.33) | * |
| Low SDI |  | | | | | | | | |
|  | slope1 | 0.67(0.52,0.81) | * |  | 0.83(0.71,0.95) | * |  | 0.91(0.77,1.04) | * |
|  | slope2 | 1.11(1.08,1.15) | * |  | 1.16(1.12,1.21) | * |  | 1.17(1.12,1.22) | * |
|  | slope3 | 1.61(1.28,1.94) | * |  | 1.69(1.34,2.05) | * |  | 1.65(0.85,2.45) | * |
|  | slope4 | 1.08(1.04,1.12) | * |  | 1.23(1.19,1.28) | * |  | 1.21(1.17,1.25) | * |
