# Supplementary material for: Global, regional and national burden of polycystic ovary syndrome: historical trends from 1990 to 2021 and projections to 2035
Source: Front Endocrinol (Lausanne). 2026 Apr 1;17:1662823. doi: 10.3389/fendo.2026.1662823 (PMC13079045; doi:10.3389/fendo.2026.1662823)
Supplement: Supplementary file 9 [file DataSheet4.doc]

**Table S4. Correlation between SDI and disease burden across regions and countries, and EAPC.**

| Characteristics | Incidence | |  | Prevalence | |  | DALYS | |
| --- | --- | --- | --- | --- | --- | --- | --- | --- |
|  | R | P |  | R | P |  | R | P |
| 21 regions-SDI | 0.4145 | ＜0.001 |  | 0.4133 | ＜0.001 |  | 0.4156 | ＜0.001 |
| 204 countries-SDI | 0.4480 | ＜0.001 |  | 0.4499 | ＜0.001 |  | 0.4546 | ＜0.001 |
