# Supplementary material for: Global, regional and national burden of polycystic ovary syndrome: historical trends from 1990 to 2021 and projections to 2035
Source: Front Endocrinol (Lausanne). 2026 Apr 1;17:1662823. doi: 10.3389/fendo.2026.1662823 (PMC13079045; doi:10.3389/fendo.2026.1662823)
Supplement: Supplementary file 10 [file DataSheet5.doc]

**Table S5.SDI-related health inequality slope index and relative concentration index for the global burden of polycystic ovary syndrome, 1990 and 2021.**

|  | ASIR | ASPR | ASDR |
| --- | --- | --- | --- |
| Inequality Slope Index |  |  |  |
| 1990 | 62.04(49.86, 74.22) | 1588.26(1281.30, 1895.22) | 14.24(11.45, 17.02) |
| 2021 | 77.47(60.91, 94.03) | 1973.77(1556.06, 2391.49) | 17.70(14.00, 21.41) |
| Relative Concentration Index |  |  |  |
| 1990 | 0.25(0.19, 0.29) | 0.24(0.19, 0.29) | 0.25(0.19, 0.30) |
| 2021 | 0.21(0.16, 0.25) | 0.20(0.16, 0.24) | 0.21(0.16, 0.25) |
